# Two Invaders Instead of One: The True Identity of Species under the Name *Ceracis cucullatus* (Coleoptera: Ciidae)

**DOI:** 10.1371/journal.pone.0072319

**Published:** 2013-08-28

**Authors:** Caio Antunes-Carvalho, Cristiano Lopes-Andrade

**Affiliations:** 1 Programa de Pós-Graduação em Entomologia, Departamento de Entomologia, Universidade Federal de Viçosa, Viçosa, Minas Gerais, Brazil; 2 Departamento de Biologia Animal, Universidade Federal de Viçosa, Viçosa, Minas Gerais, Brazil; Swedish University of Agricultural Sciences, Sweden

## Abstract

The Neotropical obligate fungivorous beetle *Ceracis cucullatus* (Mellié) has attracted attention of coleopterists due to the increasing number of records of populations in Africa. Although its disjunct populations have been interpreted as a cohesive taxonomic unity, previous comparisons between African and Neotropical specimens revealed differences in their external morphology, causing uncertainty about the true unity of the species. Here, we compare the external morphology of specimens named *Cer. cucullatus* from several localities of the Neotropical, Palearctic, Afrotropical, Afrotemperate and Oriental regions. As results, we reverse three previous junior synonymies of *Cer. cucullatus*, proposing *Cer. lamellatus* (Pic) and *Cer. tabellifer* (Mellié), both reinstated status and new combinations, as separate species. We also propose *Enn. bilamellatum* Pic as a new synonym of *Cer. tabellifer*. In face of these taxonomic changes, we identify *Cer. tabellifer* as the actual invasive species on African lands, instead of *Cer. cucullatus* as was previously accepted. Then, through historical records gathered from scientific collections and literature, and through examination of recently collected specimens from South Africa and Brazil, we provide data on host fungi and geographic distribution of *Cer. tabellifer*. Based on these data, we discuss possible explanations to the successful invasion of *Cer. tabellifer* in Africa and elsewhere and its potential threat to native faunas of ciids. This study helps to fulfil an old gap in the literature on biological invasions, with considerably more studies on predatory species, disease vectors or potential pests of agricultural crops, than on non-pest fungivorous organisms.

## Introduction

**Figure 1:**
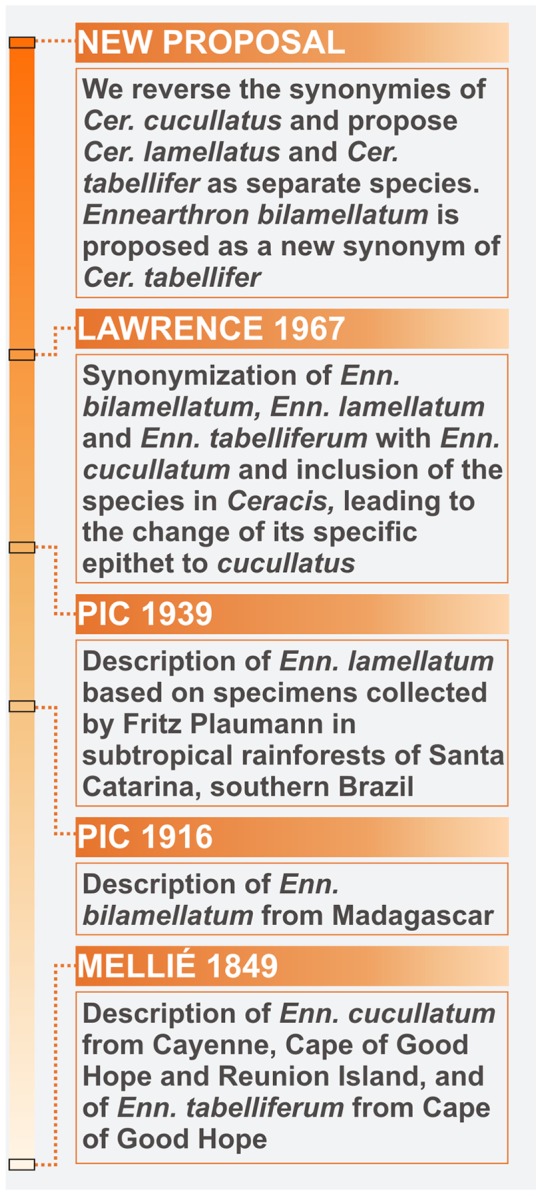
Timeline of the taxonomy of species previously under the name *Ceracis cucullatus* (Mellié).

*Ceracis cucullatus* (Mellié) belongs to Ciidae (Coleoptera: Tenebrionoidea), a family of small obligate fungivorous beetles that live and breed in polypore basidiomes worldwidely. *Ceracis cucullatus* was originally described as *Ennearthron cucullatum* by Mellié in 1849 [1], based on specimens from Cayenne (French Guiana), Cape of Good Hope (South Africa) and Reunion Island, and subsequently transferred to *Ceracis* Mellié by Lawrence [2] (Fig. 1). It names the *cucullatus* species-group, which currently comprises *Cer. bicornis* (Mellié), *Cer. cassumbensis* Antunes-Carvalho & Lopes-Andrade, *Cer. cucullatus* and *Cer. navarretei* Antunes-Carvalho & Lopes-Andrade. It also encompasses the names *Ennearthron tabelliferum* Mellié, *Enn. Bilamellatum* Pic and *Enn. lamellatum* Pic, junior synonymies of *Cer. cucullatus*. These synonymies were proposed by Lawrence [2] who argued that they were described as new based primarily on differences in size and development degree of male pronotal projections. Such secondary sexual characteristics exhibit a wide variation in size and sometimes even in form, as they have allometric growth. It is common to find male ciids with either conspicuous or weak secondary characteristics coexisting in a single population. This phenotypic plasticity may hamper the delimitation and identification of species, and may lead to the proposition of synonymies.

As *Enn. Bilamellatum* was described from Madagascar, *Enn. Tabelliferum* from South Africa and *Enn. lamellatum* from Brazil (Fig. 1), the synonymization of these with *Cer. cucullatus* led it to be considered a broadly distributed species. The records of populations in areas beyond its native range are old and have accumulated over the years, attracting the attention of coleopterists. Populations have been found in Italy, France and Britain [3], [4], [5], although they appear not to be free-living in these countries (note that another invasive species cited for Britain [5] is *Cis bilamellatus* Wood, a very distinct species with almost the same specific epithet of *Ennearthron bilamellatum*). Various records of populations in islands are also known, such as in Galapagos, in the Pacific Ocean, and Reunion, Mauritius, Seychelles and Aldabra, in the western Indian Ocean [2], [6], [7]. As most species of *Ceracis* inhabits the Neotropical region, and given the absence of other *Ceracis* species in Africa, non-Neotropical populations of *Cer. cucullatus* have been interpreted as introduced species [2], [7].

The most reliable way to distinguish ciid species is combining traditional information of the external morphology with that of male abdominal terminalia. High morphological stability and specificity of male abdominal terminalia is observed not only in ciids, but in many other animals, especially arthropods [8] and it is largely explained by its rapid evolutionary divergence among phylogenetically related species [9]. For this reason, the taxonomic importance and use of the morphology of abdominal terminalia, mainly of genitalia, is widespread in animal taxonomy. Despite this, in the taxonomy of subtropical and tropical Ciidae the use of male abdominal terminalia to distinguish species was consolidated mostly over the last decade, during which several new ciid species have had their descriptions largely supported by the morphology of this structure [10], [11], [12], [13], [14], [15]. Although the disjunct populations named *Cer. cucullatus* have been interpreted as a cohesive taxonomic unity, our preliminary comparisons between African and Neotropical specimens revealed inconsistencies in their external morphology, including male abdominal terminalia, leading us to doubt on the conspecificity of populations under this name.

In the present study we aim to evaluate the status of disjunct populations under the name *Cer. cucullatus*. Thus, we analysed and compared the external morphology, including male abdominal terminalia, of populations from several localities of the Neotropical, Palearctic, Afrotropical, Afrotemperate and Oriental regions. As results, we reverse the synonymies of *Enn. bilamellatum, Enn. Lamellatum* and *Enn. Tabelliferum* with *Cer. cucullatus*, propose *Cer. lamellatus* (Pic) and *Cer. tabellifer* (Mellié), both reinstated status and new combinations, as separate species and propose *Enn. bilamellatum* as a new synonym of *Cer. tabellifer*. We also redescribe *Cer. cucullatus*, *Cer. lamellatus* and *Cer. tabellifer*. Additionally, based on data of host fungi and historical records of *Cer. tabellifer*, we conduct a wide discussion on its successful invasion in Africa and other regions.

## Material and Methods

### Comparisons

**Figure 2:**
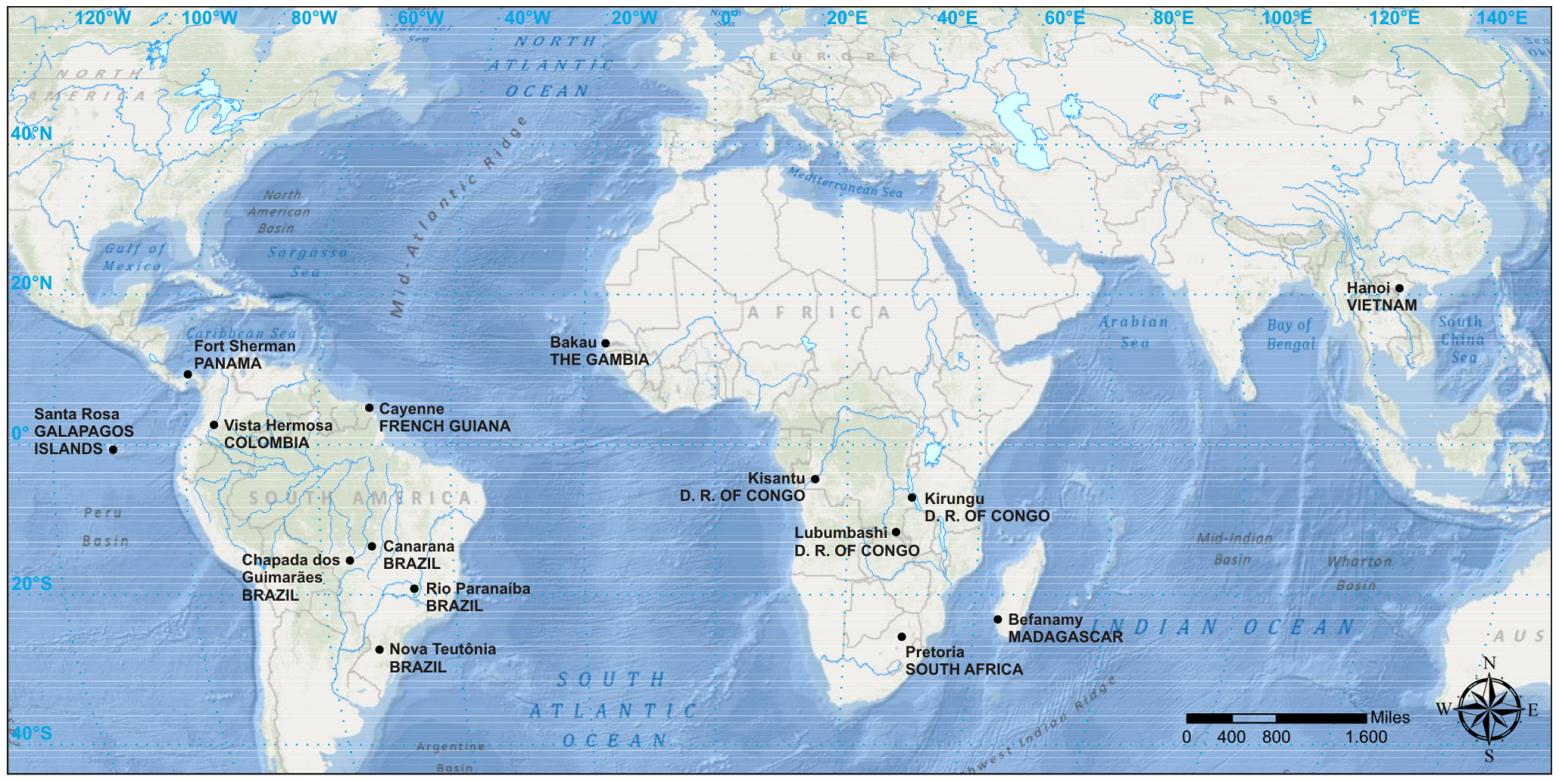
Geographic location of dissected male specimens.

We examined and compared the external morphology of a representative number of specimens originally named *Cer. cucullatus* (Fig. 1) from localities in the Neotropical, Palearctic, Oriental, Afrotropical and Afrotemperate regions, including a number of islands of the western Indian Ocean. We also extracted and dissected the male abdominal terminalia of representative specimens from these regions (Fig. 2) and carefully compared their morphology. Comparison, examination, measurement and dissection of specimens were made under a Zeiss Stemi 2000-C stereomicroscope or a Zeiss Axiolab microscope. Terms used here for external morphology, including male abdominal terminalia, are explained and discussed by Lopes-Andrade and Lawrence [16].

### Pictorial documentation

**Figure 3:**
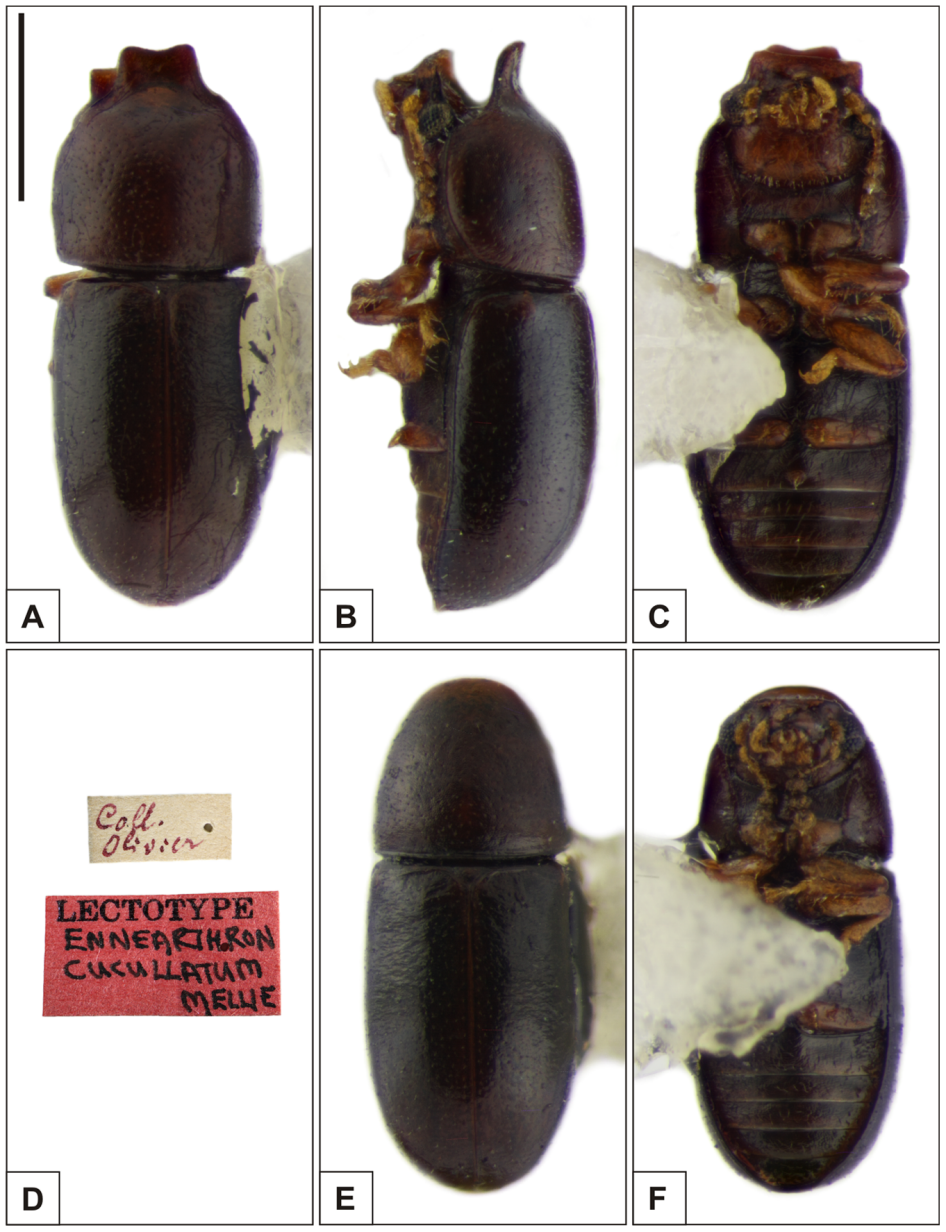
Habitus of *Ceracis cucullatus* (Mellié). A–C Lectotype male, (A) dorsal view, (B) lateral view, (C) ventral view, (D) label data. E–F Paralectotype female, (E) dorsal view, (F) ventral view. All figures are in the same scale, except for labels. Scale bar  = 0.5 mm.

**Figure 4:**
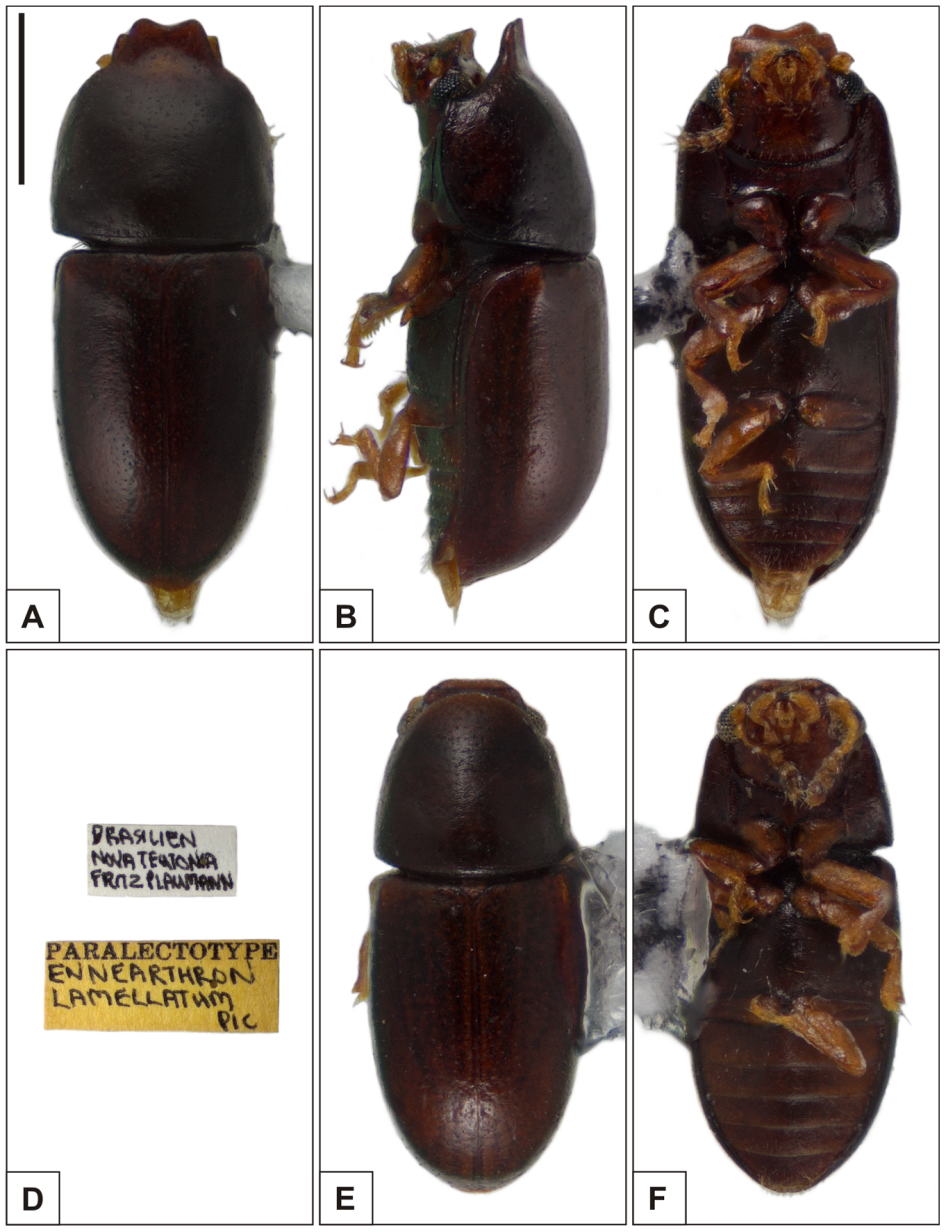
Habitus of *Ceracis lamellatus* (Pic). A–C Paralectotype male, (A) dorsal view, (B) lateral view, (C) ventral view, (D) label data. E–F Paralectotype female, (E) dorsal view, (F) ventral view. All figures are in the same scale, except for labels. Scale bar  = 0.5 mm.

**Figure 5:**
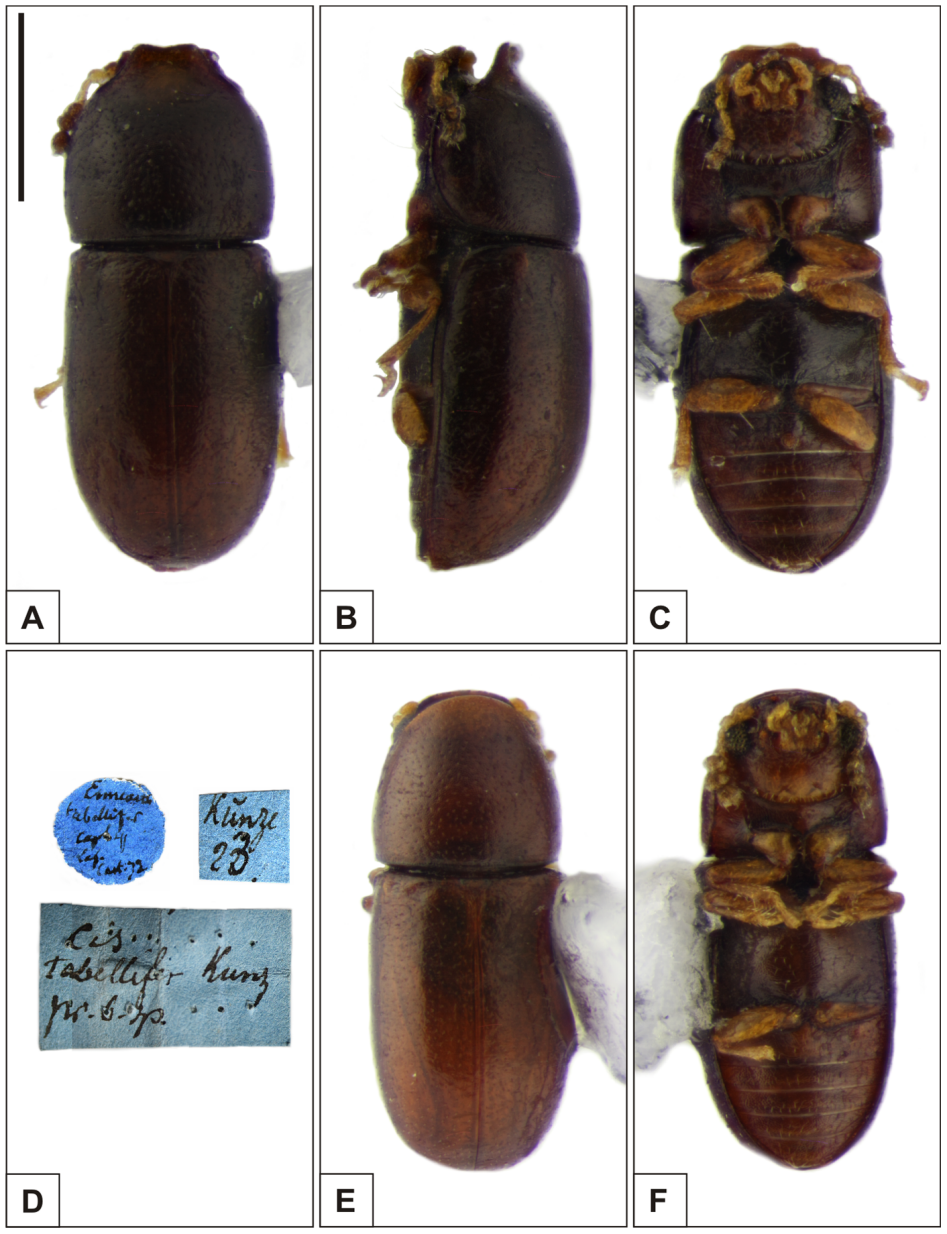
Habitus of *Ceracis tabellifer* (Mellié). A–C Lectotype male, (A) dorsal view, (B) lateral view, (C) ventral view, (D) label data. E–F Paralectotype female, (E) dorsal view, (F) ventral view. All figures are in the same scale, except for labels. Scale bar  = 0.5 mm.

**Figure 6:**
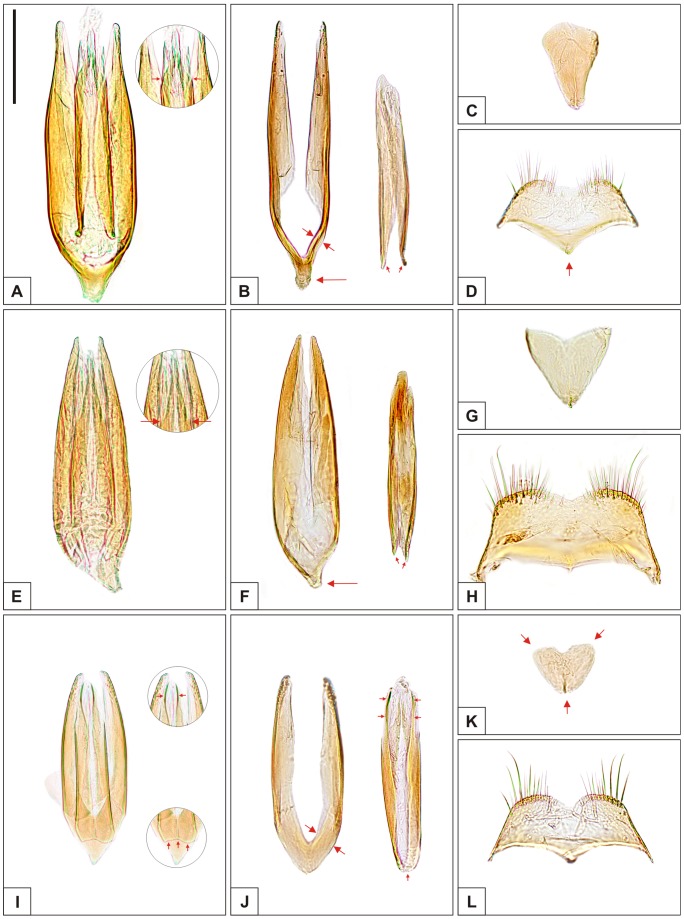
Male abdominal terminalia of *Ceracis cucullatus* (Mellié), *Cer. lamellatus* (Pic) and *Cer. tabellifer* (Mellié). Whole mount preparations of aedeagus (A, E, I), tegmen and penis (B, F, J), basal piece (C, G, K) and eighth sternite (D, H, L) of (A–D) *Ceracis cucullatus* (Mellié), (E–H) *Cer. lamellatus* (Pic) and (I–L) *Cer. tabellifer* (Mellié). Some features of the penis are better visualized in whole mount preparations of aedeagus and therefore are highlighted in figures A, E and I. Arrows indicate morphological characteristics important to distinguish the species, as follows: (A) Angulation point from which the margins of penis converge abruptly toward the apex. (B) Tegmen with basal portion narrowed, distinct (large arrow); basolateral margins narrow (medium-sized arrows); penis with basal portion opened, leaving the lower limits of the lateral margins disconnected (small arrows). (D) Eighth sternite with anterior margin distinctly produced at middle, its apex conspicuously beyond the anterolateral angles. (E) Angulation point from which the margins of penis converge abruptly toward the apex. (F) Tegmen with basal portion narrowed, distinct (large arrow); penis with basal portion opened (small arrows). (I) Above, penis with apical portion forming well sclerotized arcs (horizontal small arrows); below, basal portion of penis closed, membranous (vertical small arrows). (J) Tegmen with basolateral margins thick (large arrows); penis with basal portion closed (vertical small arrow) and apical portion forming well sclerotized arcs (horizontal small arrows). (K) Basal piece small, semicircular or subtriangular, with rounded angles. All figures are in the same scale. Scale bar  = 0.1 mm.

**Figure 7:**
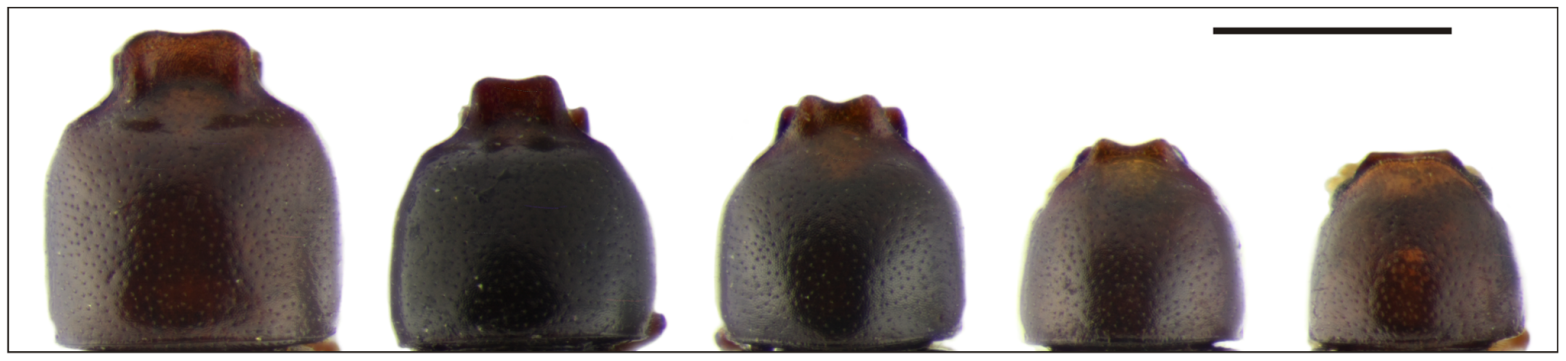
Morphological variability in *Ceracis tabellifer* (Mellié). Differences in development degree of the pronotal projections between males of different sizes. All figures are in the same scale. Scale bar  = 0.5 mm.

**Figure 8:**
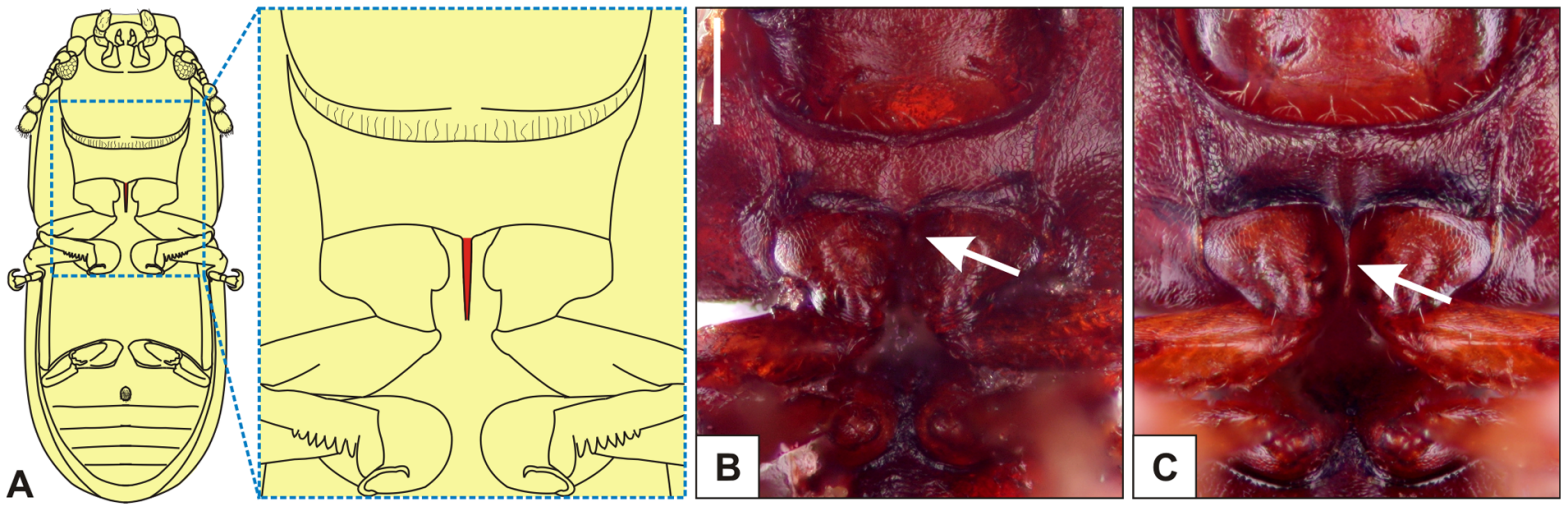
Prosternum of *Ceracis tabellifer* (Mellié). (A) Diagram of an adult *Cer*. *tabellifer* in ventral view showing the prosternal process (in red). In the figures B and C the white arrows indicate the reduction of the prosternal process in a specimen from Vietnam (B), compared to a specimen from Democratic Republic of Congo (C). The images are excessively reddish because they were taken using a transmitted light source. The figures are in the same scale. Scale bar  = 0.1 mm.

Digital photographs of adult specimens were taken with a Canon EOS 1000D digital camera attached to a Zeiss Stemi 2000-C stereomicroscope. Photographs showing the prosternum were taken under a Zeiss Axiolab A1 compound microscope equipped with the same camera. Final images were the result of joining 20 to 50 photomicrographs at different focal depths using the image stacking software Zerene Stacker (v1.04). The names *Cer. cucullatus*, *Cer. lamellatus* and *Cer. tabellifer* used from now on refer to these species in the senses and combinations here proposed (Fig. 1), unless otherwise specified. For the sake of organization, we show images of specimens of the type series and labels of the three species consecutively (Figs. 3, 4, 5), a plate comparing sclerites of their male abdominal terminalia (Fig. 6), a comparison of development degree of pronotal projections in *Cer. tabellifer* (Fig. 7) and variation of morphology of the prosternum in the species (Fig. 8). Images and redescriptions of *Cer. cucullatus* and *Cer. tabellifer* are based on the respective male lectotype, here designated, and those of *Cer. lamellatus* on a male paralectotype. The specimen chosen as lectotype of *Cer. lamellatus*, here designated, was examined but could not be borrowed and was therefore not pictured. The syntypes of species treated in the present work were all labelled as lectotypes or paralectotypes by John F. Lawrence, but they were not officially designated in the literature. We preferred to maintain Lawrence's labels. Whole mount preparations followed the protocol described by Lopes-Andrade [14], and photographs were taken under a Zeiss Axiolab A1 compound microscope equipped with a Zeiss Axiocam Erc5S or a Canon EOS 1000D digital camera.

### Measurements

Specimens of *Cer. cucullatus* (82 specimens), *Cer. lamellatus* (17) and *Cer. tabellifer* (90) were measured. The values provided for *Cer. cucullatus* are of the lectotype and from populations collected in Brazil (45), Panama (18) and Galapagos (18). For *Cer. tabellifer*, we measured the lectotype and specimens from Brazil (10), South Africa (26), Congo (40) and Gambia (13). Values for *Cer. lamellatus* are of paralectotypes (2) and topotypes (15). Topotypes, as used here, are specimens collected in the type locality but not labelled as paratypes [17]. Abbreviations used for measurements (in millimetres) and ratios are provided in Table 1. Range, mean and standard deviation are given for each measurement and ratio in the section on “Variation”.

**Table 1:** Abbreviations for measurements and ratios used in the section on Taxonomy.

BW	basal width of the scutellum
CL	length of the antennal club^a^
EL	elytral length^b^
EW	greatest elytral width
FL	length of the antennal funicle^c^
GD	greatest depth of the body^d^
PL	pronotal length along midline
PW	greatest pronotal width
SL	scutellum length
TL	total length^e^
GD/EW	degree of convexity
TL/EW	degree of body elongation

a From the base of the antepenultimate antennomere to the apex of the apical one.

b Median length from base of scutellum to elytral apex.

c From the base of the third antennomere to the apex of the sixth.

d From elytra to metaventrite.

e EL+PL, head not included.

### Dissected material

We dissected male specimens previously named *Cer. cucullatus*, or unidentified specimens resembling the species, from the following localities (number of dissected specimens between parentheses): Canarana (7), Chapada dos Guimarães (1), Nova Teutônia (2) and Rio Paranaíba (2) (Brazil), Vista Hermosa (1) (Colombia), Cayenne (1) (French Guiana), Fort Sherman (1) (Panama), Santa Rosa (1) (Galapagos Islands), Pretoria (1) (South Africa), Kirungu (1), Kisantu (1) and Lubumbashi (3) (Democratic Republic of Congo), Bakau (1) (Gambia), Hanoi (1) (Vietnam) and Befanamy (1) (Madagascar). Therefore, we have representative samples of male abdominal terminalia from populations located at the southern and northernmost distribution of species previously under the name *Cer. cucullatus* (Fig. 2). Lectotypes and paralectotypes were not dissected, as the Muséum national d'Histoire naturelle (Paris, France) has not granted permission to dissect type material.

### Maps and geographic distribution

**Figure 9:**
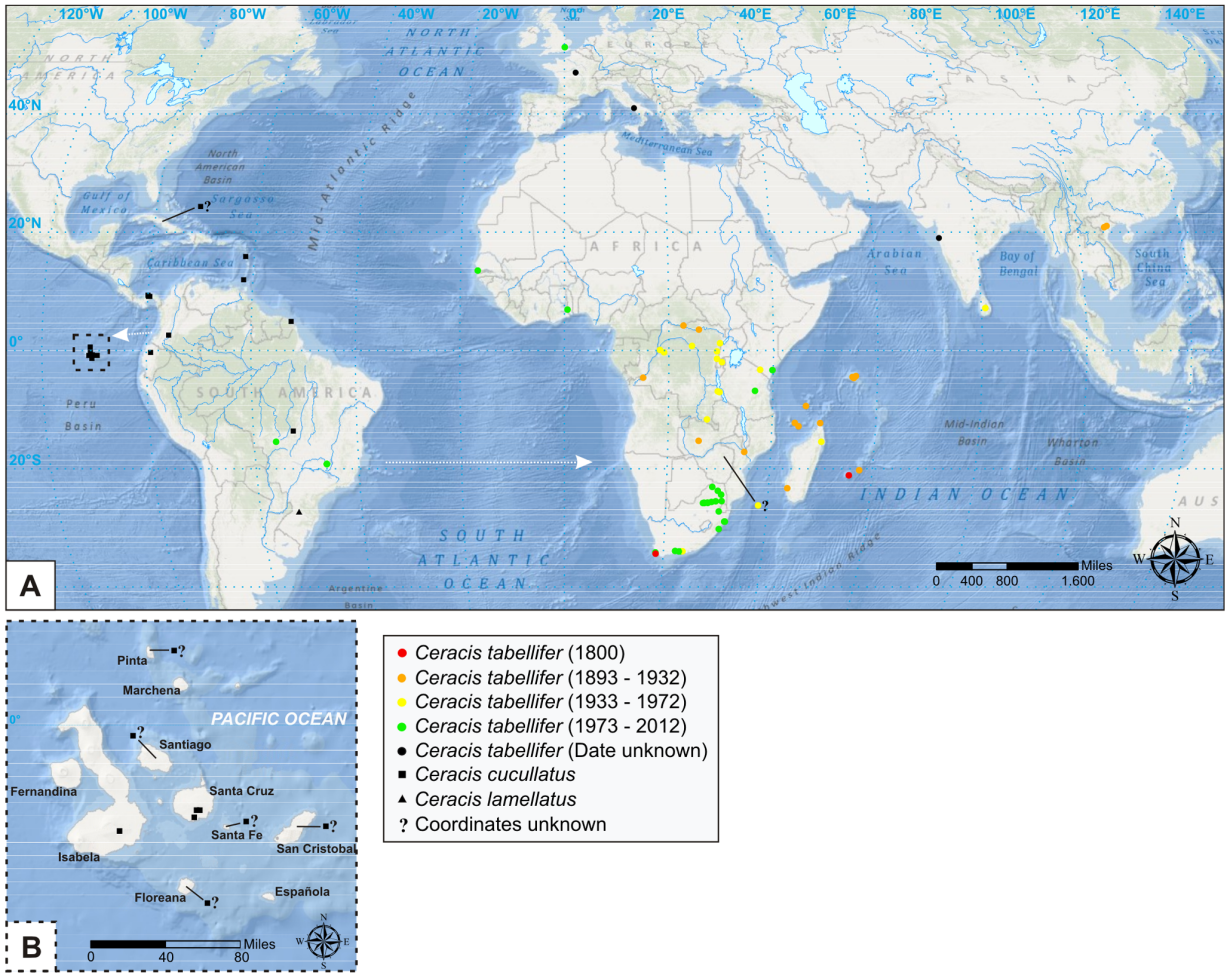
Geographic distribution of *Ceracis cucullatus* (Mellié), *Cer. lamellatus* (Pic) and *Cer. tabellifer* (Mellié). (A) Arrows indicate the possible direction of introductions of *Cer. cucullatus* and *Cer. tabellifer* in the Galapagos Islands and Africa, respectively. The records of *Cer. tabellifer* are divided into four time periods, represented by different colors. Undated records are represented by black circles (see map legend). Between 1800 and 1893, no record was found. (B) Geographic distribution of *Cer. cucullatus* in the Galapagos Islands. Records of *Cer. cucullatus* from Cuba and Galapagos Islands (Floreana, Pinta, San Cristobal, Santa Fé, Santiago), and of *Cer. tabellifer* from Zimbabwe are indicated by “?”, because it was not possible to determine the exact collection site.

Aiming to trace the geographical distribution of *Cer. cucullatus*, *Cer. lamellatus* and *Cer. tabellifer* (Fig. 9), we searched for records of these species by direct observation of labels in museum specimens and throughout literature. Dates for each record on the map were obtained from specimens' labels. For records obtained from scientific literature, we assigned the date of publication of the work providing the information. We estimated latitude and longitude coordinates by tracking localities in the online database GeoNames [18] and plotted them in a map using the software ArcGis 9.3 (ESRI, Redlands, CA, USA).

Here, invasive species refers to non-native species that establish populations and spread widely beyond the site of initial introduction [19], [20], [21]. This is not necessarily associated to environmental impact.

### Material examined

Most examined specimens were obtained in loans authorized by the following researchers and respective institutions: Bert Viklund (NHRS–Naturhistoriska Riksmuseet, Stockholm, Sweden), François Génier (CMN–Canadian Museum of Nature, Ottawa, Canada), Giulio Cuccodoro (MHNG–Muséum d'Histoire Naturelle, Geneva, Switzerland), John F. Lawrence (ANIC–Australian National Insect Collection, CSIRO Ecosystem Sciences, Canberra, Australia), Klaus-Dieter Klass (SNSD –Senckenberg Naturhistorische Sammlungen Dresden, Dresden, Alemanha), Manfred Uhlig (MFN–Museum für Naturkunde, Berlin, Germany), Marc De Meyer (KMMA–Koninklijk Museum voor Midden Afrika, Tervuren, Belgium), Riaan Stals (SANC–South African National Collection of Insects, Pretoria, South Africa), Roy Danielsson (MZLU–Museum of Zoology, Lund University, Lund, Sweden), Sônia Casari (MZUSP– Museu de Zoologia da Universidade de São Paulo, São Paulo, Brazil) and Thierry Deuve (MNHN–Muséum national d'Histoire naturelle, Paris, France). The remaining specimens are from LAPC (Cristiano Lopes-Andrade Private Collection, Viçosa, Minas Gerais, Brazil).

## Taxonomy

Based on patterns of the external morphology of adults, including male abdominal terminalia, here we (i) reinstate both *Enn. tabelliferum* and *Enn. lamellatum* as separate species, and propose the new combinations *Cer. tabellifer* and *Cer. lamellatus*, (ii) reverse the synonymy of *Enn. bilamellatum* with *Cer. cucullatus* and (iii) propose *Enn. bilamellatum* as a new synonym of *Cer. tabellifer*, as the populations from continental Africa (*Cer. tabellifer*) and Madagascar (described as *Enn. bilamellatum*) do not have consistent morphological differences. Therefore, the *cucullatus* species-group now includes the following six species:

- *Ceracis bicornis* (Mellié, 1849).
- *Ceracis cassumbensis* Antunes-Carvalho & Lopes-Andrade, 2011.
- *Ceracis cucullatus* (Mellié, 1849), **new sense.**
- *Ceracis lamellatus* (Pic, 1939), **reinstated status and new combination.**
- *Ceracis navarretei* Antunes-Carvalho & Lopes-Andrade, 2011.
- *Ceracis tabellifer* (Mellié, 1849), **reinstated status, new sense and new combination** *Ennearthron bilamellatum* Pic, 1916, **new synonym.**

Although specimens of *Cer. cucullatus*, *Cer. lamellatus* and *Cer. tabellifer* are very similar morphologically, we consider the differences in the morphology of their male abdominal terminalia, shown here for the first time, as sufficient to allow their recognition as separate species. *Ceracis tabellifer* has a comparatively smaller tegmen (Figs. 6I–J) with basal portion subtriangular or rounded and basolateral margins thick (Fig. 6J, large arrows). Additionally, its penis has the basal portion membranous (Figs. 6I–J, vertical small arrows) and apical portion forming a well sclerotized arc (Figs. 6I–J, horizontal small arrows). In *Cer. cucullatus* and *Cer. lamellatus*, the basal portion of tegmen is narrowed (Figs. 6B, F, large arrows), as well as the basolateral margins in *Cer. cucullatus* (Fig. 6B, medium-sized arrows). Both species are similar in the basal portion of penis (Figs. 6B, F, small arrows), but the apical portion is different. The penis in *Cer. cucullatus* is narrowed along the apical one-fourth of its length (Fig. 6A), and in *Cer. lamellatus* it is narrowed at the apical third (Fig. 6E). Moreover, in *Cer. cucullatus* the eighth sternite has the anterior margin distinctly produced at middle (Fig. 6D, arrow), while it is weakly projected or almost straight in *Cer. lamellatus* (Fig. 6H). The basal piece also differs between species. The one of *Cer. tabellifer* has round angles (Fig. 6K, arrows) and is smaller than those of *Cer*. *cucullatus* (Fig. 6C) and *Cer. lamellatus* (Fig. 6G).

The redescriptions of *Cer. cucullatus*, *Cer. lamellatus* and *Cer. tabellifer* are provided below, as well as an identification key for adult males of *Ceracis* of the *cucullatus* species-group.

### *Ceracis cucullatus* (Mellié, 1849), new sense

(Figures 3, 6A–D, 9)

#### Diagnosis

Tegmen (Figs. 6A–B) with basal portion narrow (Fig. 6B, large arrow); basolateral margins narrow (Fig. 6B, medium-sized arrows). Penis (Figs. 6A–B) elongate, subcylindrical; basal portion opened, leaving the lower limits of the lateral margins disconnected (Fig. 6B, small arrows); lateral margins narrow, subparallel at the basal three-fourths, abruptly converging at the beginning of the apical one-fourth (Fig. 6A, arrows); apical portion weakly sclerotized. Basal piece (Fig. 6C) elongate, subtriangular, nearly one-third the length of the tegmen and 1.5 times as long as wide. Eighth sternite (Fig. 6D) with anterior margin distinctly produced forward and angulate at middle, its apex conspicuously beyond the anterolateral angles (Fig. 6D, arrow).

#### Lectotype (Figs 3A–C)

**Measurements** (in mm): TL 1.49, PL 0.64, PW 0.56, EL 0.85, EW 0.58, GD 0.51. Ratios: PL/PW 1.13, EL/EW 1.48, EL/PL 1.33, GD/EW 0.89, TL/EW 2.59. **Body** elongate, subcylindrical; dorsal and ventral surfaces mostly dark reddish brown; appendices yellowish brown. **Head** barely visible from above; dorsal surface smooth, with a small salience at middle; frontoclypeal ridge produced forward, transversely concave, its anterior margin weakly emarginated at middle, the anterior edge with a row of setae along it. **Eyes** coarsely facetted, with minute slender yellowish setae emerging from the intersection between ommatidia. Each **antenna** with nine antennomeres; length of antennomeres (in mm) as follows (from base to apex): 0.06, 0.04, 0.03, 0.02, 0.02, 0.02, 0.04, 0.05, 0.06 (left antenna measured; FL 0.09 mm, CL 0.15 mm, CL/FL 1.67); each antennomere of the club bearing several sparse slender setae and four conspicuous sensillifers positioned at its upper portion. **Pronotum** with subparallel sides; lateral margins narrow, visible from above only for the posterior corners; anterior edge projected forwards forming a quadrangular plate, slightly emarginated at middle; anterolateral angles obtuse, not produced; disc with relatively fine, single, uniformly distributed punctation; interstices in between punctures from one to two puncture-widths; vestiture consisting of yellowish decumbent minute seta. **Scutellum** small, subtriangular, glabrous; BW 0.08 mm, SL 0.05 mm. **Elytra** with humeral calli; lateral margins subparallel at the basal two-thirds, then abruptly converging to the apex; only the anterior angles visible from above; punctation single, very fine, confused, denser than pronotal punctation; vestiture consisting of minute decumbent yellowish setae; interstices in between punctures smooth and shiny, shallowly rugose. **Ventral sclerites** with most of their surfaces granulate. **Prosternum** in front of coxae shallowly concave longitudinally and transversely convex; surface beside coxae weakly concave; prosternal process laminate, almost as long as coxae. **Metaventrite** moderately convex, subglabrous, with sparse slender setae; punctation not observed; discrimen indiscernible. Each **protibia** with the apex expanded; outer apical angle rounded and bearing a row of spines. **Abdominal ventrites** bearing several slender setae, longer than those on dorsal surface; punctation shallow and sparse; lengths of abdominal ventrites (from base to apex, at the longitudinal midline) as follows (in mm): 0.19, 0.07, 0.08, 0.08, 0.07; first abdominal ventrite with a basal width of 0.48 mm and bearing a circular sex patch located postered of center, with a transverse diameter of 0.04 mm. **Male abdominal terminalia** (of specimens compared to the type; Figs 6A–D) as follows: Tegmen (Figs 6A–B) three to four times as long as wide, twice as wide as penis, and about 1.5 times as long as the greatest width of the eighth sternite; basal portion narrowed, distinct (Fig. 6B, large arrow); basolateral margins narrow (Fig. 6B, medium-sized arrows); lateral margins subparallel at the basal two-thirds of its length, and then slightly angulate at the beginning of the apical third; apex of each lateral margin narrow, with a few sensilla. Penis (Figs 6A–B) elongate, subcylindrical; basal portion opened ( = not membranous), leaving the lower limits of the lateral margins disconnected (Fig. 6B, small arrows); lateral margins narrow, subparallel at the basal three-fourths, abruptly converging at the beginning of the apical one-fourth (Fig. 6A, arrows); apical portion narrow and weakly sclerotized. Basal piece (Fig. 6C) subtriangular, elongate, nearly one-third the length of the tegmen and 1.5 times as long as wide. Eighth sternite (Fig. 6D) with posterior margin slightly emarginated at middle; posterior corners rounded, bearing bristles; lateral margins diverging from the posterior to the anterior portion; anterior margin distinctly produced and angulate at middle, its apex conspicuously beyond the anterolateral angles (Fig. 6D, arrow).

#### Variation

**Males**, measurements in mm (n = 55, including the lectotype): TL 0.94–1.53 (1.29±0.14), PL 0.43–0.68 (0.57±0.07), PW 0.40–0.56 (0.49±0.05), EL 0.48–0.85 (0.72±0.07), EW 0.41–0.58 (0.49±0.04), GD 0.38–0.51 (0.44±0.04). Ratios: PL/PW 1.00–1.29 (1.16±0.06), EL/EW 1.12–1.55 (1.45±0.06), EL/PL 1.03–1.47 (1.27±0.09), GD/EW 0.85–0.95 (0.89±0.02), TL/EW 2.21–2.76 (2.59±0.11). Body varying from dark brown or dark reddish brown to black. Frontoclypeal ridge and anterior edge of pronotum varying from strongly projected in large males to weakly or not produced in small ones. In the last case, the frontoclypeal ridge and anterior margins of pronotum are rounded, making the smallest males morphologically similar to females. In males from Midwest Brazil, the pronotal projection is usually projected forward, not upward. The body size of specimens from northern South America and Galapagos Islands is greater than those of Midwest Brazil. Size difference is also noticeable in male abdominal terminalia, which is longer in insular populations. **Females**, measurements in mm (n = 27): TL 1.00–1.44 (1.19±0.12), PL 0.38–0.64 (0.46±0.06), PW 0.39–0.55 (0.46±0.04), EL 0.63–0.89 (0.73±0.06), EW 0.40–0.59 (0.49±0.04), GD 0.36–0.51 (0.44±0.04). Ratios: PL/PW 0.91–1.28 (1.01±0.07), EL/EW 1.38–1.56 (1.48±0.04), EL/PL 1.25–1.80 (1.60±0.10), GD/EW 0.86–0.92 (0.89±0.02), TL/EW 2.23–2.67 (2.41±0.09). Females (Figs 3E–F) are usually smaller than males, with frontoclypeal ridge, lateral and anterior margins of pronotum rounded, not produced. Head with dorsal surface devoid of prominences. Abdominal sex patch absent.

#### Host fungi

The following records of host fungi were taken from labels, mainly from specimens collected in Panama, and updated consulting the online database of Index Fungorum (http://www.indexfungorum.org): *Ganoderma applanatum* (Ganodermataceae), *Rigidoporus* sp. (Meripilaceae), *Gloeoporus thelephoroides* (Meruliaceae), *Coriolopsis caperata*, *Earliella scabrosa*, *Hexagonia hydnoides*, *Pycnoporus sanguineus* and *Trichaptum sector* (Polyporaceae).

#### Distribution (Fig. 9)

From southern Mexico to Midwest Brazil. Also in the Galapagos Islands (possibly introduced).

#### Type material

**French Guiana**: Lectotype (MNHN), here designated\Coll. Olivier [handwritten]\[red label] LECTOTYPE [printed] Ennearthron cucullatum Mellie [handwritten]\(see Fig. 3D); 2 paralectotypes (MNHN)\[blue circular label] Enneartho cucullatum [handwritten] [words difficult to interpret]\[yellow label] PARALECTOTYPE [printed] Ennearthron cucullatum Mellie [handwritten]\.

#### Additional material

**Brazil**: 50 specimens (LAPC)\BRASIL: MT Canarana; “Faz. Santa Marta” 22.xii.2008 C.M. Mews leg.\. **Colombia**: 1 specimen (LAPC)\Colombia: Meta, Vista Hermosa, Vda. La Reforma PNN La Macarena iv.2010 [printed]. 2–3 [handwritten] leg J.L. Contreras\2°37′52′′N 75°44′10.3′′W 269 m\; 1 specimen (MFN)\53174 [printed]\lobifer M. [handwritten]\[green label] Hist.-Coll. (Coleoptera), Nr.53174 (1.Ex.) Cis lobifer Moritz Columb., Moritz, Zool. Mus. Berlin\; 1 specimen (MFN)\[green label] Hist.-Coll. (Coleoptera), Nr.53174 (6.Ex.) Cis lobifer Moritz Columb., Moritz, Zool. Mus. Berlin.\. **Cuba**: 1 specimen (MNHN)\Cuba, Ennearthron cucullatum [handwritten]. **Ecuador**: 2 specimens (ANIC)\ECUAD: Pichincha Rio Palenque, 47 km.s Sto.Domingo May 18–29, 1975\J.F. Lawrence Lot No. [printed] 4055 [handwritten]\A. Forsyth S.&J. Peck collectors\Rigidoporus\; 1 specimen (ANIC)\ECUAD: Pichincha Rio Palenque, 47 km.s Sto.Domingo July 20–30, 1975[handwritten]\J.F. Lawrence Lot No. [printed] 4062 [handwritten]\A. Forsyth S.&J. Peck collectors\Rigidoporus\. **French Guiana**: 7 specimens (MNHN)\Cayenne [handwritten]\[blue label] MUSEUM PARIS, Collection Léon Fairmaire, 1906\Ennearthron cucullatum [handwritten]. **Galapagos Islands**: 1 specimen (CMN)\ECU: Galap; St. Cruz 2KmN Bellavista 360 m, guavathicket 14.V-13.VII.85, S&J Peck Agricultural area, FIT\[yellow label] CMN 108\; 2 specimens (ANIC)\ECU: Galap; St. Cruz 2KmN Bellavista 360 m, guavathicket 14.V-13.VII.85, S&J Peck Agricultural area, FIT\; 1 specimen (CMN)\ECU: Galapagos Isabela, Sto. Tomas 4–15.III.89, 330 m humidforest, FIT Peck&Sinclair, 89–100\[yellow label] CMN 112\; 1 specimen (CMN)\ECU: Galapagos SantaCruz, Sta. Rosa., Scalesia [underlined] zone bracketfungi 5.II.89, S. Peck, 89–100\[yellow label] CMN 111\; 1 specimen (CMN)\ECU: Galapagos SantaCruz, Sta. Rosa., Scalesia [underlined] zone bracketfungi 5.II.89, S. Peck, 89–100\[yellow label] CMN 109\; 1 specimen (CMN)\ECU: Galapagos SantaCruz, 5 KmN Pto. Ayora, trans.z. III.89, bracketfungi S. Peck 89–192\[yellow label] CMN 110\; 2 specimens (ANIC)\Santa Cruz Is., Galapagos II-1964\J.F. Lawrence Lot. [printed] 1293 [handwritten]\R.L.Usinger Coll. [handwritten]\ex Ganoderma applanatum\. **Guadeloupe**: 1 specimen (MNHN)\[blue label] MUSEUM PARIS, GUADELOUPE, Env. De Trois-Riviéres, LEO DUFAU 1904 [printed]\Ceracis cucullatus (Mellie) [handwritten] 1969 J.F. Lawrence\. **Panama**: 2 specimens (ANIC)\Barro Colorado Is. CANAL ZONE July [printed] 6 [handwritten] 1969 [printed]\J.F. Lawrence Lot. [printed] 2791 [handwritten]\Rigidoporus sp.\; 3 specimens (ANIC)\Barro Colorado Is. CANAL ZONE Feb. 14, 1968\J.F. Lawrence Lot. [printed] 2412 [handwritten]\Polyporus conchoids\; 2 specimens (ANIC)\Fort Sherman Canal Zone Panama, IV-2-67\J.F. Lawrence Lot. [printed] 2079 [handwritten]\Trametes corrugata\; 1 specimen (ANIC)\Barro Colorado Is. CANAL ZONE Feb. 23, 1968\J.F. Lawrence Lot. [printed] 2474 [handwritten]\Trametes cirrifer\; 1 specimen (ANIC)\Barro Colorado Is. CANAL ZONE Feb. 23, 1968\J.F. Lawrence Lot. [printed] 2263 [handwritten]\Trametes cirrifer\; 1 specimen (ANIC)\Barro Colorado Is. CANAL ZONE Aug. [printed] 6 [handwritten] 1969 [printed]\J.F. Lawrence Lot. [printed] 2996 [handwritten]\Trametes corrugata [printed]\; 1 specimen (ANIC)\Madden Dam CANAL ZONE VII-18-1969\J.F. Lawrence Lot. [printed] 2903 [handwritten]\Polyporus sanguineus\; 1 specimen (ANIC)\Madden Dam CANAL ZONE VII-18-1969\J.F. Lawrence Lot. [printed] 2905 [handwritten]\Polyporus hydnoides\; 4 specimens (ANIC)\Madden Dam CANAL ZONE VII-18-1969\J.F. Lawrence Lot. [printed] 2908 [handwritten]\Hexagona sp.\; 1 specimen (ANIC)\Barro Colorado Is. CANAL ZONE July [printed] 25 [handwritten] 1969 [printed]\J.F. Lawrence Lot. [printed] 2961 [handwritten]\ex Polyporus sector\; 1 specimen (ANIC)\Barro Colorado Is. CANAL ZONE July [printed] 10 [handwritten] 1969 [printed]\J.F. Lawrence Lot. [printed] 2833 [handwritten]\Ganoderma sp.\; 2 specimens (ANIC)\Fort Sherman Canal Zone Panama, IV-2-67\J.F. Lawrence Lot. [printed] 2085 [handwritten]\Rigidoporus sp.\; 2 specimens (ANIC)\Barro Colorado Is. CANAL ZONE July [printed] 3 [handwritten] 1969 [printed]\J.F. Lawrence Lot. [printed] 2750 [handwritten]\Ganoderma sp.\; 2 specimens (ANIC)\Fort Sherman Canal Zone Panama, IV-2-67\J.F. Lawrence Lot. [printed] 2079 [handwritten]\Trametes corrugata\; 3 specimens (ANIC)\CANAL ZONE: 5mi. SW Fort Sherman Mas. 2, 1975 Lawrence, Erwin\J.F. Lawrence [printed] Lot 3841 [handwritten]\.

### *Ceracis lamellatus* (Pic, 1939), reinstated status and new combination

(Figures 4, 6E–H, 9)

#### Diagnosis

Tegmen (Figs 6E–F) with basal portion narrow, differentiated (Fig. 6F, large arrow); lateral margins converging gradually to apex. Penis (Figs 6E–F) elongate; basal portion opened (Fig. 6F, small arrows); lateral margins narrow, subparallel at the basal one third, converging gradually along the second third of its length, and abruptly converging at the beginning of the apical third (Fig. 6E, arrows). Basal piece (Fig. 6G) subtriangular, nearly one-third the length of the tegmen and about as wide as long. Eighth sternite (Fig. 6H) with posterior corners somewhat angulate.

#### Male paralectotype (Figs 4A–C)

**Measurements** (in mm): TL 1.68, PL 0.70, PW 0.65, EL 0.98, EW 0.65, GD 0.60. Ratios: PL/PW 1.08, EL/EW 1.5, EL/PL 1.39, GD/EW 0.92, TL/EW 2.58. **Body** elongate, convex; elytra, apex of pronotum, prosternum, mesoventrite and abdominal ventrites reddish brown, remainder of pronotum and metaventrite dark reddish brown; appendices yellowish brown. **Head** barely visible from above; dorsal surface smooth, with a small salience at middle; frontoclypeal ridge produced forward, transversely concave, its anterior margin weakly emarginated at middle, the anterior edge with a row of setae along it. **Eyes** coarsely facetted; some minute slender yellowish setae emerging from the intersection between ommatidia. Each **antenna** with nine antennomeres; length of antennomeres (in mm) as follows (from base to apex): 0.06, 0.05, 0.04, 0.03, 0.03, 0.03, 0.05, 0.05, 0.06 (right antenna measured; FL 0.13 mm, CL 0.16 mm, CL/FL 1.23); each antennomere of the club bearing several sparse slender setae and four conspicuous sensillifers positioned at its upper portion. **Pronotum** with subparallel sides; lateral margins narrow, visible from above only for the anterior corners; anterior edge projected forwards forming a quadrangular plate, slightly emarginated at middle (see the section on “Variation”); anterolateral angles obtuse, not produced; disc with relatively fine, single, uniformly distributed punctation; interstices in between punctures from 1.50 to three puncture-widths; vestiture consisting of yellowish decumbent minute seta. **Scutellum** small, triangular, glabrous; BW 0.13 mm, SL 0.08 mm. **Elytra** with humeral calli; lateral margins subparallel at the basal half, then gradually converging to the apex; only the anterior angles visible from above; punctation single, very fine, confused, denser than pronotal punctation; vestiture consisting of minute decumbent yellowish setae; interstices in between punctures smooth and shiny, shallowly rugose. **Ventral sclerites** with most of their surfaces granulate. **Prosternum** in front of coxae shallowly concave longitudinally and transversely convex; surface beside coxae weakly concave; prosternal process laminate, almost as long as coxae. **Metaventrite** moderately convex, subglabrous, with sparse slender setae; punctation indiscernible; discrimen not visible. Each **protibia** with the apex expanded; outer apical angle rounded and bearing a row of spines. **Abdominal ventrites** bearing several slender setae, longer than those on dorsal surface; punctation indiscernible; length of the abdominal ventrites (from base to apex, at the longitudinal midline) as follows (in mm): 0.20, 0.06, 0.06, 0.06, 0.09; first abdominal ventrite with a basal width of 0.56 mm and bearing a circular sex patch located postered of center, with a transverse diameter of 0.04 mm. **Male abdominal terminalia** (of specimens compared to the paralectotype; Figs 6E–H) as follows: Tegmen (Figs 6E–F) three to about four times as long as wide, about 1.6 to twice as wide as penis, and about 1.3 to 1.4 times as long as the greatest width of the eighth sternite; basal portion narrowed (Fig. 6F, large arrow); lateral margins converging gradually to apex; apex of each lateral margin narrow, with a few sensilla. Penis (Figs. 6E–F) elongate; basal portion opened ( = not membranous) (Fig. 6F, small arrows); lateral margins narrow, subparallel at the basal one third, converging gradually along the second third of its length, and abruptly converging at the beginning of the apical third (Fig 6E, arrows); apical portion narrowed and moderately sclerotized. Basal piece (Fig. 6G) subtriangular, nearly one-third the length of the tegmen and about as wide as long. Eighth sternite (Fig. 6H) with posterior margin barely emarginated at middle; posterior corners somewhat angulate, bearing bristles; lateral margins diverging from the posterior to the anterior portion; anterior margin weakly projected at middle, almost straight.

#### Variation

**Males**, measurements in mm (n = 15, including the paralectotype): TL 1.53–1.80 (1.60±0.07), PL 0.60–0.78 (0.67±0.05), PW 0.58–0.70 (0.63±0.03), EL 0.86–1.03 (0.93±0.04), EW 0.60–0.68 (0.63±0.02), GD 0.48–0.66 (0.57±0.04). Ratios: PL/PW 0.98–1.16(1.06±0.05), EL/EW 1.38–1.55 (1.46±0.05), EL/PL 1.21–1.58 (1.39±0.10), GD/EW 0.76–0.98 (0.90±0.05), TL/EW 2.42–2.67 (2.52±0.07). Body varying from dark reddish brown to reddish brown; pronotum and elytra sometimes with the same coloration, but the pronotum being usually the darkest. Frontoclypeal ridge and anterior edge of pronotum strongly projected in the largest males and weakly produced in the smallest ones. **Females**, measurements in mm (n = 2, including a paralectotype): TL 1.38–1.48 (1.43±0.07), PL 0.48–0.50 (0.49±0.02), PW 0.53–0.58 (0.55±0.04), EL 0.90–0.98 (0.94±0.05), EW 0.60–0.63 (0.61±0.02), GD 0.55–0.55 (0.55±0). Ratios: PL/PW 0.87–0.90 (0.89±0.02), EL/EW 1.50–1.56 (1.53±0.04), EL/PL 1.89–1.95 (1.92±0.04), GD/EW 0.88–0.92 (0.90±0.03), TL/EW 2.29–2.36 (2.33±0.05). Females (Figs. 4E–F) are usually smaller and darker than males, with frontoclypeal ridge, lateral and anterior margins of pronotum rounded, not produced. Head with dorsal surface devoid of prominences. Abdominal sex patch absent.

#### Host fungi

Unknown.

#### Distribution (Fig. 9)

Known only from Nova Teutônia, southern Brazil.

#### Type material

**Brazil**: Lectotype (MNHN), here designated\BRASILIEN Nova Teutonia FritzPlaumann [handwritten]\[red label] LECTOTYPE [printed] Ennearthron lamellatum Pic [handwritten]\; 6 paralectotypes (MNHN)\BRASILIEN Nova Teutonia FritzPlaumann [handwritten]\[yellow label] PARALECTOTYPE [printed] Ennearthron lamellatum Pic [handwritten]\(see Fig. 4D).

#### Additional material

**Brazil**: 3 specimens (ANIC)\Nova Teutonia Sta. Catarina BRAZIL [printed] XI [handwritten] -1964 FritzPlaumann [printed]\; 12 specimens (MZUSP)\4 [handwritten] 196 Brasilien Nova Teutonia 27°11′B52°23′L Fritz Plaumann 300 500 m [printed]\.

### *Ceracis tabellifer* (Mellié, 1849), reinstated status, new combination and new sense

*Ennearthron bilamellatum* Pic 1916: 20, **new synonym** (Figures 5, 6I–L, 7, 8, 9).

#### Diagnosis

Tegmen (Figs 6I–J) with basal portion subtriangular or rounded; basolateral margins thick (Fig. 6J, large arrows). Penis (Figs. 6I–J) with basal portion closed, membranous (Fig. 6I–J, vertical small arrows); apical portion forming well sclerotized arcs (Fig. 6I–J, horizontal small arrows). Basal piece (Fig. 6K) small, semicircular or subtriangular with rounded angles, one-fourth the length of the tegmen and about 1.2 times as wide as long. Eighth sternite (Fig. 6L) with anterior margin weakly projected at middle, but apex at the same height of the anterolateral angles.

#### Lectotype (Figs 5A–C)

**Measurements** (in mm): TL 1.40, PL 0.55, PW 0.55, EL 0.85, EW 0.55, GD 0.50. Ratios: PL/PW 1.00, EL/EW 1.55, EL/PL 1.55, GD/EW 0.91, TL/EW 2.55. **Body** elongate, reasonably robust, subcylindrical; pronotum and ventral surfaces mostly dark reddish brown; elytra reddish brown; appendices yellowish brown. **Head** barely visible from above; dorsal surface smooth, with a small salience at middle; frontoclypeal ridge produced forward, transversely concave, its anterior margin weakly emarginated at middle, the anterior edge with a row of setae along it. **Eyes** coarsely facetted; minute slender yellowish setae emerging from the intersection between ommatidia. Each **antenna** with nine antennomeres; length of antennomeres (in mm) as follows (from base to apex): 0.06, 0.04, 0.03, 0.02, 0.02, 0.01, 0.04, 0.04, 0.05 (left antenna measured; FL 0.08 mm, CL 0.13 mm, CL/FL 1.62); each antennomere of the club bearing several sparse slender setae and four conspicuous sensillifers positioned at its upper portion. **Pronotum** with subparallel sides; lateral margins narrow, visible from above only for the anterior corners; anterior edge projected forwards forming a plate, slightly emarginated at middle (see the section on “Variation”); anterolateral angles obtuse, not produced; disc with relatively fine, single, uniformly distributed punctation; interstices in between punctures from 1.25 to two puncture-widths; vestiture consisting of yellowish decumbent minute seta. **Scutellum** small, subtriangular, punctate, glabrous; BW 0.08 mm, SL 0.04 mm. **Elytra** with humeral calli; lateral margins subparallel at the basal second third, then abruptly converging to apex; only the anterior angles visible from above; punctation single, very fine, confused, denser than pronotal punctation; vestiture consisting of minute decumbent yellowish setae; interstices in between punctures smooth and shiny, shallowly rugose. **Ventral sclerites** with most of their surfaces granulate. **Prosternum** in front of coxae shallowly concave longitudinally and transversely convex; surface beside coxae weakly concave; prosternal process laminate, almost as long as coxae. **Metaventrite** moderately convex, subglabrous, with sparse slender setae; punctation very shallow and sparse, almost imperceptible; discrimen indiscernible. Each **protibia** with the apex expanded; outer apical angle rounded and bearing a row of spines. **Abdominal ventrites** bearing several slender setae, longer than those on dorsal surface; punctation shallow and sparse; length of the abdominal ventrites (from base to apex, at the longitudinal midline) as follows (in mm): 0.17, 0.06, 0.07, 0.08, 0.08; first abdominal ventrite with a basal width of 0.48 mm and bearing a circular sex patch located postered of center, with a transverse diameter of 0.04 mm. **Male abdominal terminalia** (of specimens compared to the type; Figs. 6I–L) as follows: Tegmen (Figs. 6I–J) about three times as long as wide, about twice as wide as penis and 1.2 times as long as the greatest width of the eighth sternite; basal portion subtriangular; basolateral margins thick (Fig. 6J, large arrows); lateral margins subparallel at their basal two-thirds, angulate at the beginning of the apical third and then slightly curved to apex; apex of each lateral margin converging inwards, bearing several sensilla. Penis (Figs. 6I–J) elongate, subcylindrical; basal portion closed, membranous (Figs 6I–J, vertical small arrows); apical portion forming well sclerotized arcs (Figs. 6I–J, horizontal small arrows). Basal piece (Fig. 6K) small, semicircular or subtriangular and with rounded angles, one-fourth the length of the tegmen and about 1.2 times as wide as long. Eighth sternite (Fig. 6L) with posterior margin slightly emarginated at middle; posterior angles rounded, bearing bristles; lateral margins diverging; anterior margin weakly projected at middle, but apex at the same height of the anterolateral angles.

#### Variation

**Males**, measurements in mm (n = 62, including the lectotype): TL 1.00–1.78 (1.39±0.15), PL 0.38–0.73 (0.57±0.07), PW 0.40–0.66 (0.54±0.05), EL 0.63–1.08 (0.82±0.09), EW 0.43–0.69 (0.55±0.05), GD 0.36–0.61 (0.49±0.05). Ratios: PL/PW 0.78–1.16 (1.06±0.06), EL/EW 1.11–1.62 (1.48±0.07), EL/PL 1.09–2.00 (1.44±0.13), GD/EW 0.81–0.96 (0.88±0.03), TL/EW 2.13–2.74 (2.52±0.10). Body varying from dark brown to dark reddish brown, sometimes almost black. Frontoclypeal ridge and anterior edge of pronotum strongly projected in the largest males and weakly or not produced in the smallest ones (Fig. 7). In the smallest males, the frontoclypeal ridge and anterior margins of pronotum are rounded, making them morphologically similar to females. Specimens from invasive populations usually have body and male abdominal terminalia larger than the ones from autochthonous Neotropical populations. However, despite also having a longer male abdominal terminalia, some specimens that inhabit the tropical African savannah have a body size similar to the Neotropical ones. We have also identified two curious morphological variations in specimens from Hanoi, Vietnam: the lack of prosternal process (Fig. 8B, arrow; compare to Figs. 8A, C) and each antenna with eight antennomeres, instead of nine (see the section on “Has *Ceracis tabellifer* a Neotropical or an Afrotropical origin?”). **Females**, measurements in mm (n = 28): TL 1.11–1.61 (1.30±0.13), PL 0.41–0.59 (0.48±0.05), PW 0.41–0.61 (0.49±0.05), EL 0.70–1.03 (0.81±0.08), EW 0.45–0.65 (0.53±0.05), GD 0.40–0.61 (0.48±0.05). Ratios: PL/PW 0.95–1.05 (0.99±0.02), EL/EW 1.44–1.61 (1.52±0.04), EL/PL 1.58–1.86 (1.69±0.07), GD/EW 0.86–0.95 (0.89±0.03), TL/EW 2.33–2.59 (2.43±0.07). Females (Figs. 5E–F) are usually smaller than males, with frontoclypeal ridge, lateral and anterior margins of pronotum rounded, not produced. Head with dorsal surface devoid of prominences. Abdominal sex patch absent.

#### Host fungi

Neser [22] provided a fantastic dataset on the South African Ciidae, their host fungi and parasitoids. In that work, a total of 19 Ciidae species were reported from South Africa: *Ceracis tabellifer*, 17 species of *Cis* Latreille and one species of *Xylographus* Mellié. A total of 55 individual fungi, as defined by Graf-Peters et al. [23], were collected. *Ceracis tabellifer* were found in 41 individual fungi and was the unique ciid species in 16. A total of 19 species of fungi were used as hosts by the ciids, and *Cer. tabellifer* occurred in 17 (here, we have not considered the records based on samples of mixed host species). Only basidiomes of *Pycnoporus sanguineus* and *Trametes cingulata* were devoid of adult *Cer. tabellifer*. The host fungi of *Cer. tabellifer* in South Africa are as follows (number of recorded occurrences between parentheses): *Fomitopsis lilacinogilva* (1), *Phaeolus schweinitzii* (2) (Fomitopsidaceae), *Ganoderma applanatum* (4), *G. lucidum* (1), *Ganoderma* sp. (1) (Ganodermataceae), *Phellinus* sp. (1) (Hymenochaetaceae), *Funalia polyzona* (1), *Trametes hirsuta* (4), *T. versicolor* (6), *Trametes* sp. (1), *Hexagonia tenuis* (1), *Lenzites elegans* (3), *Polyporus dictyopus* (1), *Trametes versicolor* (1), *Trametes* sp. (7) (Polyporaceae), *Stereum ostrea* (1) (Stereaceae), *Thelephora* sp. (1) (Thelephoraceae). Therefore, 67.5% of the records were in Polyporaceae and the remaining records in fungi of other families. Considering the criteria of Graf-Peters et al. [23], *Cer. tabellifer* can be considered a true polyphagous species in South Africa. It is worth mentioning here that this data refers only to specimens collected in basidiomes inhabited by parasitoids of Ciidae [22]. The other ciids collected there are currently under identification, but again the majority of specimens are *Cer. tabellifer* (Lopes-Andrade, pers. comm.).

The dataset on ciids of Rio Paranaíba (northern Minas Gerais, Brazil) available to us is unpublished (Lopes-Andrade, per. comm.). A total of 13 ciid species were collected: five species of *Ceracis* Mellié, three of *Cis*, one *Malacocis* Gorham, one *Strigocis* and three species of *Xylographus*. A total of 29 individual fungi with ciids, pertaining to ten host fungi species, were collected. *Ceracis tabellifer* was found in seven species of host fungi, always together with at least one more ciid species, as follows (number of records between parentheses): *Earliella scabrosa* (1), *Hexagonia hydnoides* (1), *Lenzites betulina* (1), *Pycnoporus sanguineus* (3), *Trametes villosa* (2) (Polyporaceae), *Ganoderma* sp. (1) (Ganodermataceae) and *Stereum* sp. (1) (Stereaceae). Among fungi hosting ciids, only three were devoid of *Cer. tabellifer*: *Phellinus gilvus* (Hymenochaetaceae), *Schizophyllum commune* (Schizophyllaceae) and *Pleurotus* (Pleurotaceae). Therefore, *Cer. tabellifer* can also be considered a polyphagous species in the surveyed locality of the Brazilian Cerrado.

#### Distribution (Fig. 9)

Widespread in subsaharan Africa and in several islands of the western Indian Ocean (Comoros, Mayotte, Madagascar, Reunion, Mauritius and Seychelles). Records also from Europe (Britain and France, possibly not free-living; and Italy), Asia (Sri Lanka, India and Vietnam) and Brazil (Cerrado biome: Chapada dos Guimarães and Rio Paranaíba).

#### Type material

**South Africa**: Lectotype (MNHN), here designated\[blue circular label]Ennearthro tabellifer Cap[?] 41 Caf[?] Cast. 72[?] [handwritten]\[blue label] Kunze 28 [handwritten]\[blue label] Cis tabellifer Kunz pr. b. sp. [handwritten]\(see Fig. 5D).

**Additional material. Brazil**: 11 specimens (LAPC)\BRASIL: MT Chapada dos Guimarães i.2004 leg. C. Ribas & R. Campos\; 6 specimens (LAPC)\BRASIL: MG Rio Paranaíba 12.xii.2011 leg. N.F. Resende\; 8 specimens (LAPC)\BRASIL: MG Rio Paranaíba 14.xii.2011 leg. N.F. Resende\; 1 specimen (LAPC)\BRASIL: MG Rio Paranaíba 17.xii.2011 leg. N.F. Resende\; 1 specimen (LAPC)\BRASIL: MG Rio Paranaíba 24.xii.2011 leg. N.F. Resende\; 1 specimen (LAPC)\BRASIL: MG Rio Paranaíba 26.xii.2011 leg. N.F. Resende\; 11 specimens (LAPC)\BRASIL: MG Rio Paranaíba 05.i.2012 leg. N.F. Resende\. **Democratic Republic of Congo**:140 specimens (93 KMMA, 47 LAPC)\MUSÉE DU CONGO Elisabethville [printed] -1932 [handwritten] De Loose [printed]\; 1542 specimens (1253 KMMA, 289 LAPC)\MUSÉE DU CONGO Elisabethville [printed] -1932 [printed or handwritten] De Loose [printed] Ch. 10 [or Ch. 19, Ch. 33, Ch. 34, Ch. 39, Ch. 40, Ch. 41, Ch. 42, Ch. 44, Ch. 45, Ch. 46, Ch. 47, Ch. 49; handwritten]\; 2 specimens (KMMA)\MUSÉE DU CONGO Eala [printed] -V-1936 [handwritten] J. Ghesquière [printed] 2639 [handwritten]\; 2 specimens (KMMA)\MUSÉE DU CONGO Eala [printed] -XI-1936 [handwritten] J. Ghesquière [printed]\; 1 specimen (KMMA)\MUSÉE DU CONGO Eala [printed] -XI-1936 [handwritten] J. Ghesquière [printed]\[green label] ex Rigidoporus [handwritten]\; 33 specimens (29 KMMA, 4 LAPC)\MUSÉE DU CONGO [printed] Kisantu 1925 (R.P. Vanderyst) sur polypore [handwritten]\; 10 specimens (6 KMMA, 4 LAPC)\[green label] Récolté sur [printed] Polypore [handwritten]\MUS. ROY. AFR. CENTR. [printed] Bas. Congo: Kisantu 1925 (R.P.H. Vanderÿst) [handwritten]\; 1 specimen (KMMA)\Yangambi. 1951 C. DONIS z. [printed] 306 [handwritten]\COLL. R. MAYNÉ COM. ÉT. BOIS CONGO R. [printed] 2326 [handwritten]\COLL. MUS. TERVUREN don R. Mayné\; 1 specimen (KMMA)\MUSÉE DU CONGO [printed] Monga (Uele) III-1931 (Lebrum) ch. 2316 [handwritten]\; 2 specimen (KMMA)\MUSÉE DU CONGO [printed] Monga (Uele. Itimbiri) III-1931 (Lebrum) [handwritten]\[label with a piece of fungus, without information]\; 1 specimen (KMMA)\MUSEÉ DU CONGO Volcap Nyamlagira (Kivu) [printed] IX-1936 [handwritten] J. Ghesquière [printed] 52 53 [handwritten]\; 5 specimens (KMMA)\MUSÉE DU CONGO [printed] angodia (Uele. Itimbiri) V-1931 (Lebrum) H.2973 [handwritten]\; 1 specimen (KMMA)\MUSÉE DU CONGO [printed] angodia (Uele. Itimbiri) V-1931 (Lebrum) H.2997 [handwritten]\; 6 specimens (5 KMMA, 1 LAPC)\MUSÉE DU CONGO [printed] Flandria -IV-1935 [handwritten] J. Ghesquière [printed] 547 [handwritten]\[green label] Eclos de champignons rouges [handwritten]; 3 specimens (KMMA)\MUSÉE DU CONGO [printed] Flandria -IV-1935 [handwritten] J. Ghesquière [printed] 547 [handwritten]\; 27 specimens (22 KMMA, 5 LAPC)\Congo Belge, P.N.G Miss. H. De Saeger PFSK. 22/8, 10-VI-52 H. De Saeger 3609\; 1 specimen (KMMA)\Congo Belge: P.N.A. 30-VII-1-VIII-1955 P. Vanschuytbroeck 13.727–28\Mont Hoyo grotte Matetu; 1.160 m\; 1 specimen (KMMA)\[blue label] Dans terreau au Berlese\COLL. MUS. CONGO Kivu: T. Lubero, Biambwe riv. Lubeu, 1000 m [printed] (for) [handwritten] R.P.M.J. Célis V-1955 [printed]\; 4 specimens (KMMA)\[blue label] Récolté dans polypore [handwritten]\COLL. MUS. CONGO Katanga: Kakontwé Sar. bois. VIII-1948 N. Leleup\. 81 specimens (65 KMMA, 16 LAPC)\[blue label] Dans polypore\COLL. MUS. CONGO Baudouinville VIII-1953 H. Bomans\; 9 specimens (7 KMMA, 2 LAPC)\COLL. MUS. CONGO Tanganika: Kamsabala 1200 m. VIII-1953 H. Bomans\. **India**: 1 specimen (MHNG)\Coll. Melly [handwritten]\CucullatumDej Bonibay Mellié [handwritten]\. **Italy**: 1 specimen (MNHN)\Gallia [handwritten]\[unreadable] Exot. [handwritten]\[unreadable]cucullatum ♀ [handwritten]\; 2 specimens (MFN)\Campania Napoli Mattai 902 [handwritten]\Ennearthron [?]cuculatum [sic] [handwritten]\; 2 specimens (MFN)\Campania Napoli Mattai 902 [handwritten]\. **Kenya**: 1 specimen (MZLU)\Kenya. M. [?] [only half of the writing is visible; the label is torn] 2050 [handwritten] m T. Palm [printed]\Ciidae gen. sp. det. Mandelshtam, 2002\[blue label] LUND 263 [printed]\; 1 specimen (MZLU)\2050 [handwritten] m T. Palm Kenya, 1660 m [printed]\[blue label] LUND 269 [printed]\. **Madagascar**: 1 specimen (MNHN)\Madagascar, Amanarivo, (Sikora)\[yellow label] Type [handwritten]\[red card] TYPE\[red card] LECTOTYPE [printed] Ennearthrom bilamellatum Pic [handwritten]\Ennearthrom bilamellatum Pic [handwritten]\Ceracis cucullatus [handwritten] O. ROSE det. [printed] 2010 [handwritten]\; 1 specimen (KMMA)\Ambodivoangy [printed] VI-1959 [handwritten] (Lavage de terre) [printed] 39. [handwritten]\MUS. ROY. AFR. CENTR. Madagascar Est: Baie d' Antongil J. Vadon\. 5 specimens (MNHN)\MADAGASCAR, Befanamy, Prov. Tullan, 20.VI.1921, H. Poisson, petit polypore n.17\; 1 specimen (MNHN)\Madagascar, Diego-Suarez, Ch. Alluaud 1893\Ceracis sp. (cucullatus gp.) [handwritten] Det. J.F. Lawrence [printed]\; 1 specimen (MNHN)\Madagascar\; 1 specimen (MFN)\Type\Madagaskar\Cis laminicollis Fairm [handwritten]\coll. L.W. SchaufuB\; 7 specimens (MFN)\Madagaskar\coll. L.W. SchaufuB\. **Mayotte**: 1 specimen (MNHN)\Mayotte [handwritten]\[blue label] MUSEUM PARIS, Collection Léon Fairmaire, 1906\. **Mozambique**: 7 specimens (MNHN)\[blues label] MUSEUM PARIS, ZAMBÉZE, INHACORO, PRÉS CHEMBA, P. LESNE 1929\. **Reunion**:3 specimens (MNHN)\Bourbon, Cucullatus [handwritten]\ex Coll. Mellié [handwritten]\;1 specimen (MNHN)\Ex : Musaeo Miniszech [printed]\Cucullatus Dej. I. Bourbon [handwritten]\. **Rwanda**:156 specimens (126 KMMA, 30 LAPC)\MUSÉE DU CONGO Urundi: Masaka [printed] (1500 m) VI-193[printed]3[handwritten] A. Becquet [printed]\; 2 specimens (KMMA)\MUSÉE DU CONGO Urundi: Masaka [printed] (1500 m) VI-193[printed]3[handwritten] A. Becquet [printed]\No scolytidae [handwritten] Det. K.E. Schedi [printed]; 5 specimens (KMMA)\[blue label] s/Champignon N° 678 [handwritten]\MUSÉE DU CONGO Urundi: Masaka [printed] (1500 m) VI-193[printed]3[handwritten] A. Becquet [printed]\; 3 specimens (1 KMMA, 2 LAPC)\[green label] s/Champignon N° 678 [handwritten]\MUSÉE DU CONGO Urundi: Masaka [printed] (1500 m) VI-193[printed]3[handwritten] A. Becquet [printed]\. **South Africa**:9 specimens (6 SANC, 3 LAPC)\SOUTH AFRICA: NW Mhlabatini Kloof, Magaliesberg 25°49′S 27°19′E 12.v.2002 O.C. Neser\Adults ex bracket fungus\NATIONAL COLL OF INSECTS Pretoria, South Africa\BF#17/18 [handwritten]\; 8 specimens (6 SANC, 2 LAPC)\SOUTH AFRICA: GAU Lynnwood Glen, Pretoria 25°46′S 28°16′E 3.xi.2002 S& O.C. Neser\Adults emerged from bracket fungus *Ganoderma applanatum* BF#10/11\NATIONAL COLL OF INSECTS (SANC) Pretoria, South Africa\; 2 specimen (1 SANC, 1 LAPC)\SOUTH AFRICA: GAUTENG Rietondale Experimental Farm, Pretoria 25°43′S 28°14′E 1330 m 14.ii.2003 S. Neser\Ex bracket fungus *Phellinus* sp. on *Gleditsia triacanthos* CAESALPINIACEAE BF# 6\NATIONAL COLL OF INSECTS Pretoria, South Africa\; 10 specimens (7 SANC, 3 LAPC)\SOUTH AFRICA: NW Dome Kloof, Magaliesberg 25°45′S 27°33′E 27.vii.2003 S. Neser\Adults ex bracket fungus on fallen log of probably *Faurea saligna* (PROTEACEAE)\Ex bracket fungi *Stereum hirsutum* &*Stereum* sp. BF#1\NATIONAL COLL OF INSECTS Pretoria, South Africa\; 28 specimens (19 SANC, 9 LAPC)\SOUTH AFRICA: NW Castle Gorge, Magaliesberg 25°49′S 27°35′E 3.viii.2002 S. Neser\Ex bracket fungus *Polyporus dictyopus* on dead tree trunk BF#2\NATIONAL COLL OF INSECTS Pretoria, South Africa\; 5 specimen (4 SANC, 1 LAPC)\SOUTH AFRICA: KZN Mpisini Nature Res. 30°12′S 30°48′E 9.vii.2008 S. & O. C. Neser\Ex bracket fungus *Funalia* sp. BF#110\NATIONAL COLL OF INSECTS Pretoria, S. Afr.\; 26 specimens (18 SANC, 8 LAPC)\SOUTH AFRICA: NW Marethwane Magaliesberg 25°47′S 27°29′E 24.viii.2002 S. Neser\Adults ex bracket fungus\NATIONAL COLL OF INSECTS Pretoria, South Africa\BF# 9/16 [handwritten]\; 26 specimens (21 SANC, 5 LAPC)\SOUTH AFRICA: GAU Pretoria, Rietondale Experimental Station 25°44′S 28°13′E 12.viii.2004 S. Neser\Ex bracket fungus *Ganoderma applanatum* BF# 25\NATIONAL COLL OF INSECTS Pretoria, S. Afr.\; 10 specimens (7 SANC, 3 LAPC)\SOUTH AFRICA: NW Mhlabatini Kloof, Magaliesberg 25°49′S 27°19′E 16.vi.2002 S. Neser\Ex bracket fungus *Ganoderma* sp. on dead Croton gratissimus EUPHORBIACEAE BF#19\NATIONAL COLL OF INSECTS Pretoria, South Africa\; 8 specimens (6 SANC, 2 LAPC)\SOUTH AFRICA: NW Castle Gorge, Magaliesberg 25°49′S 27°35′E 21.iv.2002 O.C. Neser\Adults emerged from bracket fungus *Phaeolus schweintzii* BF#13\NATIONAL COLL OF INSECTS Pretoria, South Africa\; 6 specimens (4 SANC, 2 LAPC)\SOUTH AFRICA: KZN Umlalazi Nature Res Mtunzini, 28°57′S 31°46′E 13. vii.2008 R.P. Urban\Ex bracket fungus *Coriolus hirsutus* BF#87\NATIONAL COLL OF INSECTS Pretoria, South Africa\; 8 specimens (6 SANC, 2 LAPC)\SOUTH AFRICA: MPU Alkmaar, W. Nelspruit 25°27′S 30°50′E 10.ii.2008 OC Neser\Ex bracket fungus *Trametes versicolor* BF#45\NATIONAL COLL OF INSECTS Pretoria, S. Afr.\; 20 specimens (14 SANC, 6 LAPC)\SOUTH AFRICA: GAU Pretoria, Rietondale Experimental Station 25°44′S 28°13′E ix.2003 S. Neser\Ex bracket fungus *Ganoderma lunidum* BF# 27\NATIONAL COLL OF INSECTS Pretoria, S. Afr.\; 2 specimen (1 SANC, 1 LAPC)\SOUTH AFRICA: MPU Mooihoek Farm, nr. Wakkerstroom 27°13′S 30°32′E 15.vii.2008 O&S. Neser\Ex bracket fungus *Coriolus versicolor* BF# 135\NATIONAL COLL OF INSECTS Pretoria, South Africa\; 11 specimens (8 SANC, 3 LAPC)\SOUTH AFRICA: MPU Alkmaar, W. Nelspruit 25°27′S 30°50′E 10.ii.2008 OC Neser\Ex bracket fungus *Trametes* sp.BF#47\NATIONAL COLL OF INSECTS Pretoria, South Africa\; 20 specimens (14 SANC, 6 LAPC)\SOUTH AFRICA: LIMP Blouberg Mt. NW Polokwane 23°04′S 29°00′E 27.iv.2007 O.C. Neser\Adults ex bracket fungus on fallen log\Ex bracket fungus *Trametes* sp.on fallen tree trunk BF#34\NATIONAL COLL OF INSECTS Pretoria, South Africa\; 45 specimens (30 SANC, 15 LAPC)\SOUTH AFRICA: MPU Mooihoek Farm nr Wakkerstroom 27°13′S 30°32′E 15.vii.2008 O&S Neser\Ex bracket fungus *Trametes* sp.BF#113\NATIONAL COLL OF INSECTS Pretoria, South Africa\; 12 specimens (8 SANC, 4 LAPC)\SOUTH AFRICA: MPU Alkmaar, W. Nelspruit 25°27′S 30°50′E 10.ii.2008 O.C. & S. Neser\Ex bracket fungus on *Dombeya rotundifolia* BF# 68\Ex bracket fungus *Trametes* sp.BF#68\NATIONAL COLL OF INSECTS Pretoria, South Africa\; 16 specimens (11 SANC, 5 LAPC)\SOUTH AFRICA: LIMP Otter's Den, 16km frm Hoedspruit 24°24′S 30°49′E 18.vii.2008 D. van Heerden\Ex bracket fungus *Coriolus versicolor* BF# 136\NATIONAL COLL OF INSECTS Pretoria, South Africa\; 10 specimens (7 SANC, 3 LAPC)\SOUTH AFRICA: KZN Mpisini Nature Res Umkomaas, 30°12′S 30°48′E 9.vii.2008 S. & O.C. Neser\Ex bracket fungus *Coriolus hirsutus* BF# 134\NATIONAL COLL OF INSECTS Pretoria, S. Afr.\; 9 specimens (LAPC)\SOUTH AFRICA: MPU Mooihoek Farm, nr. Wakkerstroom 27°13′S 30°32′E 15.vii.2008 O&S Neser\Ex bracket fungus *Coriolus versicolor* BF#143\NATIONAL COLL OF INSECTS Pretoria, South Africa\; 4 specimen (3 SANC, 1 LAPC)\SOUTH AFRICA: GAU Wilgepoort, NE of Bronkhorstspruit 25°37′S 29°00′E 23.iii.2010 S. Neser\Ex bracket fungus # 225 on fallen tree trunk\Ex bracket fungus *Lenzites elegans* on *Acacia karroo* BF#225\NATIONAL COLL. OF INSECTS Pretoria, South Africa\; 14 specimens (11 SANC, 3 LAPC)\SOUTH AFRICA: MPU Die Hel Nature Res. nr. Loskop Dam 25°31′S 29°48′E 10.viii.2008 S. & O.C. Neser\Ex bracket fungus *Trametes* sp. BF#147\NATIONAL COLL OF INSECTS Pretoria, South Africa\; 20 specimens (15 SANC, 5 LAPC)\SOUTH AFRICA: MPU Mooihoek Farm, nr. Wakkerstroom 27°13′S 30°32′E 15.vii.2008 O&S Neser\Ex bracket fungus *Stereum ostrea* BF#144\NATIONAL COLL OF INSECTS Pretoria, South Africa\; 4 specimen (3 SANC, 1 LAPC)\SOUTH AFRICA: MPU Nelspruit 25°29′S 30°59′E 14.viii.2009 D van Heerden\Ex bracket fungus #178 on *Acacia sieberiana* var *woodii*\Ex bracket fungus *Trametes* sp. BF# 178\NATIONAL COLL OF INSECTS Pretoria, S. Afr.\; 3 specimen (2 SANC, 1 LAPC)\SOUTH AFRICA: MPU Mooihoek Farm, nr. Wakkerstroom 27°13′S 30°32′E 15.vii.2008 O&S Neser\Ex unidentified bracket fungus BF# 168\NATIONAL COLL OF INSECTS Pretoria, S. Afr.\; 15 specimens (9 SANC, 6 LAPC)\SOUTH AFRICA: KZN Twin Streams Nursery Forest, nr Mtunzini 28°57′S 31°54′E 13.vii.2008 RP Urban\Ex bracket fungus *Coriolus versicolor* BF#167\NATIONAL COLL OF INSECTS Pretoria, S. Afr.\; 3 specimen (2 SANC, 1 LAPC)\SOUTH AFRICA LIM Wesfalia Estate nr Politsi 23°44′S 30°07′E 13.x.2009 D van Heerden\Ex bracket fungus # 223 on avocado tree\Ex bracket fungus *Lenzites elegans* BF# 223\NATIONAL COLL OF INSECTS Pretoria, South Africa\; 4 specimen (3 SANC, 1 LAPC)\SOUTH AFRICA LIM Wesfalia Estate nr Politsi 23°44′S 30°07′E 13.x.2009 D van Heerden\Ex bracket fungus # 220 on avocado tree\Ex bracket fungus *Lenzites elegans* BF# 220\NATIONAL COLL OF INSECTS Pretoria, South Africa\; 4 specimen (3 SANC, 1 LAPC)\SOUTH AFRICA LIM Wesfalia Estate nr Politsi 23°44′S 30°07′E 13.x.2009 D van Heerden\Ex bracket fungus # 218 on avocado tree\Ex bracket fungus *Coriolus versicolor* BF# 218\NATIONAL COLL OF INSECTS Pretoria, South Africa\; 4 specimen (3 SANC, 1 LAPC)\SOUTH AFRICA WCAPE Prince Alfred's Pass N of Knysna 33°58′S 23°09′E 5.xi.2009 S& OC Neser\Ex bracket fungus # 214 on fallen tree trunk\Ex bracket fungus *Lenzites elegans* BF# 214\NATIONAL COLL OF INSECTS Pretoria, South Africa\; 3 specimen (2 SANC, 1 LAPC)\SOUTH AFRICA WCAPE Montagu Pass N of George 33°54′S 22°24′E 4.xi.2009 S& OC Neser\Ex bracket fungus # 211 on *Brachylaena* neriifolia\Ex bracket fungus *Coriolus hirsutus* BF# 211\; 5 specimen (4 SANC, 1 LAPC)\SOUTH AFRICA WCAPE Montagu Pass N of George 33°54′S 22°24′E 4.xi.2009 S& OC Neser\Ex bracket fungus *Coriolopsis polyzona* BF# 202\NATIONAL COLL OF INSECTS Pretoria, South Africa\; 2 specimen (1 SANC, 1 LAPC)\SOUTH AFRICA WCAPE Montagu Pass N of George 33°54′S 22°24′E 4.xi.2009 S& OC Neser\Ex bracket fungus *Coriolus versicolor* On fallen tree trunk BF# 197\NATIONAL COLL OF INSECTS Pretoria, South Africa\; 1 specimen (MZLU)\RSA: Cape Prov. Nature's Valley, at Groot Rivier 33°58′S. 23°33′ E. 15–17.X.1994 loc.21 leg. R. Danielsson\Ciidae gen. sp. Det. Mandelshtam, 2002\[blue label] LUND 286 [printed]\; 1 specimen (MZLU)\S. Afr. Cape Prov Tzitzikama Forest. Stormsrivierpiek 13.I.51 No 137\Swedish South Africa Expedition 1950–1951 Brinck-Rudebeck\[blue label] LUND 284 [printed]\; 6 specimens (SNSD)\[blue label] Cell. Maerkel [printed] [?] [handwritten, information not understood]\Staatl. Museum für Tierkunde Dresden\; 1 specimen (SNSD)\[blue label] Cell. Maerkel [printed] [?] [handwritten, information not understood]\Staatl. Museum für Tierkunde Dresden\Cis tabellifer Cap. [handwritten]\; 1 specimen (SNSD)\Peers Cave, Fish hoek, C.T. Cape P., South Africa 26.12.95, leg. R. Predel\; 2 specimens (MNHN)\tabelliferum, Kunse Mell -, Cap bon spei. [handwritten]\Ex Coll REITTER\; 1 specimen (MFN)\tabelliferkunze, cap. [handwritten]\Coll. L.W. Schaufuss\tabelliferum Mellié, Cap bom spei [handwritten]\; 1 specimen (MHNG)\Tabellifer Kuntze, Cap. B.E. Kuntze Mellié [handwritten]\; 5 specimens (NHRS)\Cap. B. Sp\Kunze [handwritten]\; 5 specimens (MNHN)\[small square blue label, without writings]\tabellifer Caffrar KZ[Kunze] [handwritten]\Ex. Coll. REITTER [printed]\. **Sri Lanka**: 2 specimens (MHNG)\CEYLAN Central [printed], Hanguranketa 27.I.70 750 m [handwritten], MUSSARD BESUCHET LÖBL\Ceracis cucullatus (Mell) [handwritten] det. J.F. Lawrence [printed]\. **Tanzania**: 34 specimens (27 KMMA, 7 LAPC)\[blue label] dans Polypore sur Cupressus\COLL. MUS. CONGO Tanganyika Terr.: Kilimanjaro, N. de Marangu, verst. S.E., 1700 m. 16-VII-1957\Mission Zoolog. I.R.S.A.C. en Afrique orientale (P. Basilewsky et N. Leleup)\; 1 specimen (LAPC)\TANZANIA Rubeho Mts. at Ipondelo vil., 6°49′51″S 36°34′30″E, 23.xii.2011 1982 m, sift. 14, V. Grebennikov\. **The** **Gambia**: 6 specimens (MZLU)\Gambia: Bakau 6–26.XI.1984 leg. T. Palm\Ceracis cucullatus Mellié [handwritten] C.Lopes-Andrade det. 2007 [printed]\[blue label] LUND 406 [printed]; 7 specimens (MZLU)\Gambia: Bakau 6–26.XI.1984 leg. T. Palm\Ceracis cucullatus Mellié [handwritten] C.Lopes-Andrade det. 2007 [printed]\[blue label] LUND 243 [printed]. **Togo**: 4 specimens (MHNG)\TOGO PALIME, Forêt de Klouto, 20-24-IV-74 S. Vit\Ceracis sp. [handwritten] J.F. Lawrence [printed]\. **Vietnam**: 3 specimens (MNHN)\MUSEUM PARIS, TONKIN, RÉG DE HOA BINH, A DE COOMAN 1927\;32 specimens (MNHN)\3171. [handwritten]\Hanoi [handwritten]\[blue label] MUSEUM PARIS, Coll. A. CROUVELLE 1915 [printed]\. **Zambia**: 11 specimens (MFN)\Insel Funshi, W. Pemba, 1901. Voel Frkemv. [?] [handwritten, difficult to interpret]\[?] laminiferum[?] [handwritten]\.

### Key to adult males of *Ceracis* of the *cucullatus* species-group with secondary sexual characteristics fully developed

1 Lateral margins of pronotum rounded. First abdominal ventrite with a broad transversely oval sex patch. Tegmen with apex bearing a small excavation on each side. Known only from “Ilha da Cassumba” (Cassumba island) in Caravelas, northeastern Brazil... ***Cer. cassumbensis*** **Antunes-Carvalho & Lopes-Andrade.**

1′ Lateral margins of pronotum subparallel. First abdominal ventrite bearing a circular margined sex patch. Tegmen with apex devoid of excavations...**2.**

2 (1′) Apex of pronotum projected forward, with its anterior margin deeply emarginate forming two diverging horns, which may be circular in cross-section and narrow at apex. From Mexico to southern Brazil... ***Cer. bicornis*** **(Mellié).**

2′ Apex of pronotum projected forward, forming a raised foursquare plate, slightly emarginated at apex... **3.**

3 (2′) Apical third of tegmen with sides converging in straight line toward apex. Known only from southern Mexico and northern Brazil... ***Cer. navarretei*** **Antunes-Carvalho & Lopes-Andrade.**

3′ Sides of tegmen moderately curved or slightly angulate at the beginning of the apical third... **4.**

4 (3′) Tegmen with basal portion subtriangular or rounded; basolateral margins thick (Fig. 6J, large arrows); apex of each lateral margin pointing inward, bearing several sensilla. Penis with basal portion closed, membranous (Figs 6I–J, vertical small arrows); lateral margins forming a well sclerotized arc at the apical portion (Figs 6I–J, horizontal small arrows). Basal piece small, semicircular or subtriangular, with rounded angles (Fig. 6K). Known from the Brazilian Cerrado biome (Chapada dos Guimarães and Rio Paranaíba), western Europe, Africa, southern Asia and islands of the western Indian Ocean... ***Cer. tabellifer*** **(Mellié).**

4′ Tegmen with basal portion narrowed (Figs 6B, F, large arrows). Penis with basal portion opened, leaving the lower limits of the lateral margins disconnected (Figs 6B, F, small arrows); apical portion narrowed and weakly sclerotized, not forming an arc (Figs 6A, E, arrows). Basal piece comparatively larger, subtriangular...**5.**

5 (4′) Eighth sternite with anterior margin biconcave, producing a remarkable subtriangular protuberance that extends beyond the lower limits of the lateral margins (Fig. 6D, arrow). Tegmen with lateral margins subparallel at the basal two-thirds of its length, and then slightly angulate at the beginning of the apical third (Figs 6A–B). Penis with lateral margins subparallel at the basal three-fourths and abruptly converging at the beginning of the apical one-fourth (Fig. 6A, arrows). Basal piece elongate, nearly 1.5 times as long as wide (Fig. 6C). Galapagos Islands and from Mexico to southern Brazil... ***Cer. cucullatus*** **(Mellié).**

5′ Eighth sternite with anterior margin weakly projected at middle, barely biconcave to almost straight (Fig. 6H). Tegmen with lateral margins converging gradually to apex (Figs 6E–F). Penis with lateral margins subparallel at the basal one-third, converging gradually along the second-third of its length, and abruptly converging at the beginning of the apical third (Fig. 6E, arrows). Basal piece about as wide as long (Fig. 6G). Known only from Nova Teutônia, southern Brazil... ***Cer. lamellatus*** **(Pic).**

## Discussion

The amazing morphological similarity between *Cer. cucullatus*, *Cer. lamellatus*, *Cer. navarretei* and *Cer. tabellifer* challenges the skills of any taxonomist. They are not only very similar, but there is a high intraspecific morphological variability, notably in secondary sexual characteristics expressed in males, as the pronotal projections (Fig. 7). The confused taxonomic history involving *Cer. cucullatus* and species previously synonymized with it appears to be a reflex of such scenario. And it reinforces the potential and relevant role of the comparative morphology of male abdominal terminalia for solving taxonomic problems between morphologically similar Ciidae species.

### Has *Ceracis tabellifer* a Neotropical or an Afrotropical origin?

*Ceracis tabellifer* was described in 1849 based on specimens from the Cape of Good Hope, at the southern tip of the African continent [1]. Populations may be collected throughout subsaharan Africa and we report here dozens of historical records outside the neotropics and a few records from South America (Fig. 9). At first, and considering that the Neotropical specimens is not quite typical in form compared to African populations, one might suggest that *Cer. tabellifer* is an autochthonous Afrotropical species. However, considering the absence of any other *Ceracis* on Africa and the great diversity of the genus in the New World, including all the species morphologically similar to *Cer. tabellifer*, we support the alternative explanation to the remarkable presence of *Cer. tabellifer* in subsaharan Africa and other regions: a successful invasion from the neotropics.

Among the six species that currently comprises the *cucullatus* group, three are known to be polyphagous and have a wide geographic distribution: *Cer. bicornis*, *Cer. cucullatus* and *Cer. tabellifer*. The latter two are successful invaders and individuals of their invasive populations have the largest body size and male abdominal terminalia (see sections on “Variation” of *Cer. cucullatus* and *Cer. tabellifer*), and are more frequently found than their conspecific native populations. By responses to selective pressures in new habitats or due to stochastic events [20], [24], [25], phenotypic variation between native and invasive populations is quite common and usually mentioned in works on biological invasion [26], [27], [28]. Species introduced into new habitats often face foundation events, which entails in genetically based shifts in phenotypic traits, among other effects [20], [29]. For instance, it is possible that the reduction of the number of antennomeres and drastic reduction (or even loss) of the prosternal process in the population from Hanoi, Vietnam (Fig. 8B, arrow) were a consequence of foundation effect and genetic drift. These specimens were collected almost a century ago and they are possibly a subsample of the non-indigenous fauna of *Cer. tabellifer* that occupies the African mainland. Therefore, the curious lack of prosternal process seems to be an adverse effect of inbreeding. To our knowledge, this is the first record of a ciid without that structure.

Considering (i) that the greatest morphological variability of species in the *cucullatus* group is found in the neotropics, (ii) the easternmost record of *Cer. tabellifer* (Vietnam) is a morphologically impoverished population, so we assume it might be a subsample of an African population and (iii) the absence of other *Ceracis* species in Africa, we sustain here that *Cer. tabellifer* is a Neotropical species introduced elsewhere, and not the inverse. Our arguments are strong enough to sustain it as the most robust explanation at the moment.

### *Ceracis tabellifer* and the general concept of species as metapopulation lineages

In the general concept of species, as it was treated and reinterpreted by de Queiroz [30] with the goal of creating a unified concept, species may be considered as separately evolving metapopulation lineages. According to de Queiroz [30] “metapopulation lineages do not have to be phenetically distinguishable, or diagnosable, or monophyletic, or reproductively isolated, or ecologically divergent, to be species. They only have to be evolving separately from other such lineages”. This concept has serious implications to the taxonomy of invasive species, which frequently have disjunct distributions, as is the case of *Cer. tabellifer*. It shall be understood and applied with care. Adopting it without careful prior morphological and molecular analyses would lead to the separation of several invasive species into two or more species, which may be unviable and, more importantly, incorrect. In the case of our study, geographic isolation does not necessarily means that the Neotropical and Afrotropical populations of *Cer. tabellifer*, for instance, are evolving separately. We judge it is early to separate disjunct populations of *Cer. tabellifer* in different species, even considering the few morphological differences of the South American populations. Further studies incorporating molecular techniques may throw light on this issue.

### Spread and current distribution of *Ceracis tabellifer* outside the Neotropical region

During the nineteenth and twentieth century, the collection of *Cer. tabellifer* varied substantially over time and space (Fig. 9). For this reason, they do not depict the chronological advance of populations on the space, but merely a variation in collection effort. Nevertheless, the historical records we provide here may be informative and reveal how old is the colonization of non-Neotropical areas by *Cer. tabellifer*.

The first *Cer. tabellifer* recorded in African lands date back to the beginning of the nineteenth century (Fig. 9, red circles), although it is possible that its arrival occurred in the previous century or even before. Until the first half of the twentieth century, populations had been recorded at various localities from the Democratic Republic of Congo and neighbouring countries. Currently *Cer. tabellifer* is widely distributed in subsaharan Africa and possibly one of the most frequently collected ciid species in the continent (Fig. 9). Several islands at the western Indian Ocean are also inhabited by *Cer. tabellifer* [2], [7], [10] and its presence in Sri Lanka and India suggests a possible route of introduction through these islands. Considering it has reached Vietnam in the first half of the twentieth century, if not before, it would not be a surprise to find the species at the remaining Oriental region, or even at the Australotropical, Neoguinean and Neozelandic regions. In contrast to the success of this species in tropical zones and marginally tropical areas, there are few records of *Cer. tabellifer* in temperate zones. Each record in Britain and France were noted as introductions, but populations are not free-living in there [3], [31], and that is possibly the case for the records in Italy either. The supposed record from Britain was, indeed, in a fungus from Zambia [31]. Low temperatures may be an impediment to successful establishment of populations in the Palaearctic region. Besides *Cer. tabellifer*, the morphologically related and also polyphagous *Cer. cucullatus* and *Cer. bicorni*s are restricted to tropical lands too.

### Causes for success and potential threat

Examining the invasiveness of a species and predicting whether it would succeed in new habitats is still a rather complex task [32], [33], [34], [35]. However, comparisons between autochthonous and invasive species in a given ecosystem may be a way to identify features determining invasion success [19]. Despite information on life history and reproductive behaviour of tropical ciids are scarce, we hypothesized below some characteristics that may underlay, at least partially, the successful establishment and spread of *Cer. tabellifer* during the invasion process, and that continue allowing its dissemination.

Most ciids are mycetobionts, with some species exploiting several host fungi, while others are restricted to a few or even one host fungus [36]. We show that *Cer. tabellifer* is a remarkably polyphagous species in South Africa and in two surveyed localities of the Brazilian Cerrado. The available data suggest that this ciid is able to exploit more host species than native ciids in South Africa, and even more than other species of Ciidae as a whole. For comparison, see data available for host fungi of Nearctic ciids [37], [38], Neotropical [23], and for ciids from Britain, Germany, North America and Japan [39]. It is noteworthy that polyphagy is common among widely distributed species of Ciidae, as in *Cer. bicornis* [23], which also belongs to the *cucullatus* species-group, and other ciids such as *Cer. thoracicornis* (Ziegler) [37], *Cis bilamellatus* Wood [5] and *Cis creberrimus* Mellié [37], the latter two introduced in Britain [5] and Galapagos Islands [40], respectively. The diet breadth has been seen as a characteristic involved in the invasion success of animals [41], including beetles [5], [42], [43]. Polyphagy has probably contributed to the invasion success of *Cer. tabellifer*.

*Ceracis tabellifer* is a multivoltine species (as well as *Cer. cucullatus*) and may be easily collected throughout the year in South Africa, where it is the most frequently collected ciid species within its broad host range. Populations have been encountered from areas near the coast to heights of up to 1700 meters, and from urban areas and cultivated lands to endemic forest and savannah [7], [22]. The polyphagous feeding habit and the wide range of habitats where *Cer. tabellifer* is able to self-sustaining suggest a strong adaptive potential. In the Cerrado biome of Brazil, which we consider to be its native habitat, *Cer. tabellifer* was the only species of Ciidae found in all surveyed types of vegetation, from open areas to riparian forests inside the biome (Lopes-Andrade, pers. obs.). Such amplitude of supported conditions is maintained in invasive populations. Allied to it, the notable occurrence of populations in islands separated by long stretches of ocean indicate the high ability to transpose long distances, either by active migration or through human intervention. However, it is still unclear which are the actual adverse effects derived from the outstanding presence of *Cer. tabellifer* in invaded habitats, or even if it offers any threat to autochthonous species.

### Other introduced or invasive ciids

Other invasive ciid species have been recorded in several regions. The Autralasian fungivore *Cis bilamellatus* was accidently introduced in England during the nineteenth century and is currently distributed across England, Wales and southern Scotland, with further records from Ireland, Channel Island and France [5]. *Cis creberrimus*, widespread in the neotropics, is considered an introduced species in Galapagos Islands. Together with *Cer. cucullatus*, they are the unique ciid species that occupy these islands [40]. Interestingly, in the population of *Cer. cucullatus* we reared in laboratory, we have observed contamination by *Cis creberrimus* in a few basidiomes. These species coexisted in the same basidiome for a long time, where they appear to have partitioned the resource temporally. We noted that *Cer. cucullatus* had greater activity in the morning and afternoon, while *Cis creberrimus* was more active in the late afternoon. Moreover, we observed that *Cis creberrimus* is able to exploit the fungus in more advanced stages of decomposition. Temporal partitioning of a host fungus by ciids was already observed [36] and would not be a surprise to report this behaviour in the wild. *Cis fuscipes* Mellié, an abundant and widely distributed ciid in North America, has been interpreted as an invasive species in Cuba, Hawaii, Madeira, Australia and New Zealand, with several parthenogenetic populations [44], [45]. *Hadreule elongatula* (Gyllenhal) is recorded throughout the Old World and northern Africa, and the presence of this minute beetle in North America has been cited as an invasion from Europe [38], [40], although it is still uncertain. *Cis chinensis* Lawrence was described based on specimens found in commercial dried fungi exported to USA [46], [47]. Nowadays this species is free-living in Europe (Germany, Italy, France and Malta), China and Thailand (possibly autochthonous in eastern Asia), southeastern Brazil and Reunion, in the western Indian Ocean [10], [48]. Other species are possibly also invasive, as *Ennearthron victori* Lopes-Andrade & Zacaro, the unique *Ennearthron* species found in the South Hemisphere.

## Conclusion

We refute an ancient scenario in which the broadly distributed populations under the name *Cer. cucullatus* were interpreted as a cohesive taxonomic unity, and demonstrate that this ciid in fact hid for a long time two additional similar species, which we have reinstated: *Cer. lamellatus* and *Cer. tabellifer*, the latter being the true invasive *Ceracis* in Africa. Due to the morphological similarity, *Cer. cucullatus* and *Cer. tabellifer* were historically confused in the literature since they were described [6], [7], and even the descriptor was “deceived” by the similarity of these small beetles (see “Introduction”). We show that the morphology of male abdominal terminalia is the most effective way to distinguish these species.

Since the first record of *Cer. tabellifer* in Africa, and based on the number of records in the continent and on information drawn from recent field collections in South Africa, it becomes evident that this ciid overcame the sequential transitions of the invasion process successfully [19], at least in African continental lands and islands of the western Indian Ocean. However, although its biological characteristics seem to confer a great competitive ability and suggest a potential threat to native fauna, it is yet unclear whether the remarkably presence of *Cer. tabellifer* in Africa implies any ecological impact. Due to its potential to colonize new habitats and ability to transpose long distances, it is possible that *Cer. tabellifer* will spread across tropical lands, but be limited by the low temperatures of temperate zones.

Future researches should evaluate the relatedness of invasive and native populations of *Cer. tabellifer* through molecular approaches. *Ceracis tabellifer* offer an exciting opportunity to study the effects of a non-pest mycetobiont organism to native communities. Our study helps to fulfil a gap in the literature on biological invasions, with considerably more studies on predatory species, disease vectors or potential pests of agricultural crops, than on non-pest fungivorous organisms.
